# Chemical composition and antioxidant capacities of phytococktail extracts from trans-Himalayan cold desert

**DOI:** 10.1186/1472-6882-13-259

**Published:** 2013-10-07

**Authors:** Priyanka Dhar, Prabodh Kumar Bajpai, Amol Bapurao Tayade, Om Prakash Chaurasia, Ravi Bihari Srivastava, Shashi Bala Singh

**Affiliations:** 1 Defence Institute of High Altitude Research, Defence Research & Development Organisation, C/o 56 APO, Leh-Ladakh, Jammu & Kashmir, Pin 901205, India; 2 Defence Institute of Physiology and Allied Sciences, Defence Research & Development Organisation, Lucknow Road, Timarpur, Delhi, Pin 110054, India

**Keywords:** Phytococktail, Sea buckthorn, Apricot, Roseroot, Antioxidant capacity, Total polyphenol content, GC/MS analysis

## Abstract

**Background:**

Himalayan plants are widely used in traditional system of medicine both as prophylactics and therapeutics for high altitude maladies. Our aim was to evaluate the antioxidant capacities and bioactive compounds of methanol and n-hexane extracts of the phytococktail comprising of sea buckthorn (*Hippophae rhamnoides*), apricot (*Prunus armeniaca*) and roseroot (*Rhodiola imbricata*) from trans-Himalaya.

**Methods:**

The 1,1-diphenyl-2-picrylhydrazyl (DPPH), 2,2'-azinobis-(3-ethylbenzothiazoline-6-sulfonic acid) diammonium salt (ABTS) and nitric oxide (NO) radical scavenging capacities and lipid peroxidation inhibition (LPI) property of the extracts were determined. Total antioxidant power was determined by ferric reducing/antioxidant power (FRAP) assay. Total polyphenol, flavonoid, flavonol, proanthocyanidin and carotenoid were also estimated for both extracts. We have identified and quantified the phyto-chemotypes present in the methanol and n-hexane extracts by hyphenated gas chromatography/mass spectrometry (GC/MS) technique.

**Results:**

Antioxidant capacity assays using DPPH, ABTS, NO, LPI and FRAP exhibited analogous results where the phytococktail showed high antioxidant action. The phytococktail was also found to possess high quantity of total polyphenol, flavonoid, flavonol and carotenoid. A significant and linear correlation was found between the antioxidant capacities and bioactive principles. A total of 32 phyto-chemotypes were identified from these extracts by GC/MS chemometric fingerprinting. Major phyto-chemotypes identified by GC/MS were glycosides, phenylpropanoids and derivatives, terpenoids, alkaloids, phytosterols, fatty acids and esters, alkaloids and derivatives, organic acid esters and aromatic ethers with positive biological and pharmacological actions.

**Conclusion:**

The phytococktail extracts were found to contain considerable amount of diverse bioactive compounds with high antioxidant capacities. The presence of hydrophilic and lipophilic antioxidants in the phytococktail could have contributed to the higher antioxidant values. Hence, the phytococktail could be used as natural source of antioxidants to ameliorate disorders associated with oxidative stress.

## Background

In oxidative stress condition, reactive oxygen species (ROS) are produced which play a vital role in the pathogenesis of several chronic diseases [1]. Extensive epidemiological studies have been conducted to ensure that intake of botanical products is linked with a reduced risk of several chronic diseases [2] and these positive properties of the plant products have been partly ascribed to the components that possess antioxidant capacities [3-6]. The natural antioxidants obtained from botanical resources have turned out to be an interesting alternative to synthetic antioxidants due to safety concerns and limitation of usage. Bioactive compounds from plants, for example, polyphenols, phenolic acids, flavonoids, flavonols, diterpenes, tannins, phytosterols, fatty acid esters, phenylpropanoids, alkaloids, glycosides etc. have received great interest in medicinal chemistry and natural product research for their high antioxidant properties [7]. Isolation and structural elucidation of these bioactive compounds is of prime importance in natural product research to identify and evaluate the therapeutic potential of medicinal plants. Numerous extraction techniques and analytical systems have been developed for the analysis and characterization of active compounds from medicinal plants. Gas chromatography/mass spectrometry (GC/MS) has become an ideal technique for qualitative and quantitative analysis of volatile and semivolatile compounds of plant origin [8].

The high altitude region of trans-Himalayan cold desert possesses adverse climatic conditions for human survival. Sustained energy deficit, malnutrition, vitamin and mineral deficiency and metabolic disorders could occur in this unfavorable environment due to alteration in physiological function [9-11]. However, the Himalaya also has the deep underlying remedy to combat these problems in its diverse flora and fauna. Plants of high altitude Himalaya are widely used in traditional system of medicine both as prophylactics and therapeutics for high altitude maladies. In recent times, a number of herbal products have been formulated from our institute using the native plants of this region [12]. We aimed at the preparation of a phytococktail comprising of sea buckthorn (*Hippophae rhamnoides* L., Elaeagnaceae), apricot (*Prunus armeniaca* L., Rosaceae) and roseroot (*Rhodiola imbricata* Edgew., Crassulaceae) which may be capable of providing additional physiological benefits and basic nutritional requirements in these extreme climatic conditions. The selected plants are widely used in the traditional system of medicine for treatment of common ailments. The advancement in the health promoting properties has led to use them as nutritional supplements [13-16]. The bioactive phytochemical components, medicinal values, therapeutic potential and nutritional properties of these three plants have been extensively studied by previous researchers [6,17-20] and the plant parts having medicinal properties were also reported to be safe and non-toxic [21-26]. We studied the methanol (hydrophilic) and n-hexane (lypophilic) extracts of the phytococktail to measure the total antioxidant capacity because both hydrophilic and lypophilic antioxidants contribute to the total antioxidant capacity. Additionally, the relationship between bioactive compounds and antioxidant capacities of hydrophilic and lipophilic extracts of the phytococktail is still unknown. Therefore, the objective of the present study was to evaluate the antioxidant capacities of the phytococktail extracts by different assays, including DPPH, ABTS, NO, LPI and FRAP methods and their correlation with bioactive compounds present in the extracts. In addition, we performed hyphenated GC/MS analysis to identify and quantify the phyto-chemotypes present in the methanol and n-hexane extracts of the phytococktail.

## Methods

### Chemicals

1,1-diphenyl-2-picrylhydrazyl radical (DPPH^**∙**^), 2,2'-azinobis-(3-ethylbenzothiazoline-6-sulfonic acid) diammonium salt (ABTS), 2,4,6-tripyridyl-*s*-triazine (TPTZ), ferrous sulfate (FeSO_4_.7H_2_O), aluminium chloride (AlCl_3_), sodium acetate (C_2_H_3_NaO_2_), sodium carbonate (Na_2_CO_3_), potassium persulfate (K_2_S_2_O_8_), potassium chloride (KCl), ferric chloride (FeCl_3_ · 6H_2_O), sodium nitroprusside (Na_2_[Fe(CN)_5_NO] · 2H_2_O), egg yolk emulsion, sulfanilic acid (C_6_H_7_NO_3_S), naphthyethylenediamine dihydrochloride, glacial acetic acid, butylated hydroxytoluene (BHT), butylated hydroxyanisole (BHA), ascorbic acid, quercetin and catechin were purchased from Sigma-Aldrich (St. Louis, MO, USA). Folin-Ciocalteu’s phenol reagent, vanillin, hydrochloric acid, sulphuric acid, methanol, n-hexane, chloroform, ethanol and sodium carbonate were purchased from Merck Chemical Supplies (Merck KGaA, Darmstadt, Germany). All other chemicals used including solvents were of analytical grade.

### Plant materials

The flora of trans-Himalayan Ladakh region was extensively studied by previous investigators [27,28]. Based on the medicinal, nutritional and therapeutic potential, three native plants of this region *viz.* sea buckthorn (*Hipppophae rhamnoides* L. subspecies *turkestanica*, family Elaeagnaceae), apricot (*Prunus armeniaca* L., family Rosaceae) and roseroot (*Rhodiola imbricata*, family Crassulaceae) were selected to develop the phytococktail.

Sea buckthorn (*H. rhamnoides*) berries were collected from Choglamsar village of Leh, Ladakh, India [altitude 3500 m above mean sea level (MSL), latitude 34°6'38.9664" N, longitude 77°35'10.3992" E], in September, 2010. The fruits of *P. armeniaca* (apricot, halman variety) were collected from apricot field gene bank of Defence Institute of High Altitude Research, Leh (altitude 3500 m above MSL, latitude 34°8'16.119" N, longitude 77°34'19.2216" E), in September, 2010. Roots of *R. imbricata* (roseroot), were collected from the Changthang valley of trans-Himalayan region (Chang-La Top, altitude 5330 m above MSL, latitude 34°2'49.812" N, longitude 77°55'49.7778" E) of India in the month of October, 2010 after the period of senescence. All necessary permits were obtained from the concerned authorities for collection of plant materials. Collected plant specimens were carefully examined and identified by Dr. Om Prakash Chaurasia, renowned plant taxonomist and principal scientist of Medicinal and Aromatic Plant (MAP) Division of our institute. The voucher specimens of *H. rhamnoides* (HR 6-8), *P. armeniaca* (PR 4-6) and *R. imbricata* (RI 5-7) have been deposited at the institutional herbarium for future reference.

### Processing of plant materials and preparation of phytococktail

Mechanical pulping of sea buckthorn berries and apricot fruits yielded raw pulp that were 50% and 70% of the fruit weight. The pulp was then lyophilized using a Lyophilizer (Model ALPHA 2-4 LDplus, Martin Christ Gefriertrocknungsanlagen GmbH, Osterode am Harz, Germany) to obtain the dry pulp powder and stored in airtight food grade container at -80°C, till the formulation of the phytococktail. Roots of *R. imbricata* were washed thoroughly, cut into small pieces and shade dried at room temperature for 15 days. Root dry matter content (DMC) was calculated as the percentage of dry weight relative to fresh weight [DMC (%) = Sample dry weight × 100 / Sample fresh weight]. The DMC was 28-33%. Then they were finely powdered and used for extraction. The root powder was taken for extraction in 80% ethanol by Soxhlet apparatus (Borosil GlassWorks Limited, Worli, Mumbai, India). The ethanolic fraction was concentrated by rotary evaporator (Rotavapor®-210, Büchi Labortechnik AG, Flawil, Switzerland) under reduced pressure at 40°C by circulation of cold water using thermostat maintained at 4°C in order to minimize the degradation of thermolabile compounds and lyophilized to obtain dry extract.

The powdered materials of *H. rhamnoides* fruit pulp, *P. armeniaca* fruit pulp and *R. imbricata* dry root extract were taken in the ratio of 100:50:1 (mg/ml) [29-31], mixed properly and dissolved in water to get a homogenous mixture of the phytococktail. It was then lyophilized to obtain the dry cocktail. To avoid contamination, clean and sterile conditions were maintained during the whole process.

### Preparation of the phytococktail extract

Soxhlet extraction was carried out with 10 gm of ground dried phytococktail with methanol and n-hexane at 40°C. The mixture was subsequently filtered (Whatman No. 5) on a Büchner funnel, the filtrate was then evaporated to dryness under reduced pressure at 40°C and lyophilized to obtain the dry extracts. Yield of the extracts were 30% (methanol extract) and 10% (n-hexane extract) of the dry phytococktail.

### DPPH radical scavenging assay

The effect of extract on DPPH radical was determined using a previously described method [32]. A solution of 0.135 mM DPPH in methanol was prepared and 100 μl of this solution was mixed with 100 μl of the phytococktail extract. The concentration of plant extracts was 20-500 μg/ml. The reaction mixture was vortexed thoroughly and left in the dark at room temperature for 30 min. The absorbance of the mixture was measured spectrophotometrically at 517 nm. Quercetin (QR), ascorbic acid (AA) and butylated hydroxytoluene (BHT) were used as standards. The ability to scavenge DPPH radical was calculated by the following equation: DPPH radical scavenging capacity (%) = [(Abs_control_ – Abs_sample_)] / (Abs_control_)] × 100,

where, Abs_control_ is the absorbance of DPPH radical + methanol; Abs_sample_ is the absorbance of DPPH radical with sample extract or standard.

The half maximal inhibitory concentration (IC_50_) for scavengers (radical scavenging concentration _50_ or RSa_50_), the amount of antioxidant required to decrease the initial DPPH concentration by 50%, termed as efficiency concentration (EC_50_) and the effectiveness of antioxidant and radical scavenging capacity demonstrated as antiradical power (ARP) were calculated [33-36]. The RSa_50_ value was determined by plotting the scavenging capacity against the logarithm of sample concentration. The EC_50_ was calculated from the following formula:

$${\text{EC}}_{50}={\text{IC}}_{50}/\left[\text{DPPH}\right]\;\text{in}\;\text{mg}/\text{ml}.$$

The ARP was also determined as follows:

$$\text{ARP}=1/\left({\text{EC}}_{50}\times 100\right)$$

The results were expressed as ascorbic acid equivalent antioxidant capacity (AEAC) [37] using the following equation:

$$\text{AEAC}=\left({\text{IC}}_{50\left(\text{AA}\right)}/{\text{IC}}_{50\left(\text{sample}\right)}\right)\times 10^{5}$$

### ABTS radical scavenging assay

The ABTS assay was performed as described by previous investigators [38]. The stock solutions included 7 mM ABTS solution and 2.4 mM potassium persulfate (PPS) solution. The working solution was then prepared by mixing the two stock solutions in equal quantities and allowing them to react for 12 h at room temperature in dark. The solution was then diluted by mixing 1 ml ABTS^.+^ solution with 60 ml of methanol to obtain an absorbance of 0.706 ± 0.001 units at 734 nm using spectrophotometer (Spectramax M2^e^, Molecular Devices, Germany). The concentration of plant extracts was 20-500 μg/ml. Plant extracts (100 μl) were allowed to react with 100 μl of the ABTS^.+^ solution and the absorbance was taken at 734 nm after 7 min incubation at 25°C in 96 well plate. The ABTS^.+^ scavenging capacity of the extracts was compared with that of QR, AA and BHT. The scavenging percentage was calculated as follows:

ABTS radical scavenging capacity (%) = [(Abs_control_ – Abs_sample_)] / (Abs_control_)] × 100,

where, Abs_control_ is the absorbance of ABTS radical + methanol; Abs_sample_ is the absorbance of ABTS radical with sample extract or standard.

The RSa_50_, EC_50_, ARP and AEAC values were also calculated as described in the previous section.

### Inhibition of lipid peroxidation

A modified thiobarbituric acid reactive species (TBARS) assay was used to measure the lipid peroxide formed using egg yolk homogenates as lipid-rich media [39]. Briefly, readymade egg yolk emulsion was diluted to 10% v/v with 1.15% w/v KCl and mixed thoroughly. The reaction solution (400 μl) consisted of 50 μl egg yolk emulsion, 50 μl of sample solution in different concentrations (2.5-500 μg/ml), 150 μl of 20% (aqueous) trichloroacetic acid and 150 μl of 0.67% w/v thiobarbituric acid. The whole reaction solution was then vortexed thoroughly and followed by incubation at 95°C for 1 h. After cooling, equal volume of butanol was added (in case of n-hexane extract) and the mixture was centrifuged at 3000 rpm for 10 min. Absorbance of the upper layer was measured at 532 nm and percentage inhibition was calculated with the following formula:

% inhibition of lipid peroxidation = (1 - t/c) × 100, where c is the absorbance of fully peroxidized control and t is the absorbance of test sample. α-tocopherol, BHA and BHT were used as reference standards. The IC_50_ value was calculated from the regression equation between sample concentration and rate of inhibition.

### Nitric oxide radical scavenging assay

The nitric oxide radical scavenging capacity of the phytococktail extracts was determined by previously established method [40]. The reaction solution (300 μl) containing 250 μl of 10 mM sodium nitroprusside in PBS (pH 7.0) was mixed with 50 μl phytococktail extracts at different concentrations (20-500 μg/ml) and followed by incubation at 37°C for 1 h. After that, 125 μl aliquot was mixed with 125 μl Griess reagent [1 ml of sulfanilic acid reagent (0.33% prepared in 20% glacial acetic acid at room temperature for 5 min) mixed with 1 ml of naphthyethylenediamine dihydrochloride (0.1% w/v)] and the absorbance was measured at 540 nm. BHA was used as the positive standard. The scavenging percentage of nitric oxide generated was measured by comparing with the absorbance value of negative control (10 mM sodium nitroprusside and PBS) by the formula described earlier.

### Total antioxidant capacity (FRAP assay)

FRAP assay was carried out to determine the total antioxidant capacity of the phytococktail extracts [41]. The stock solutions included 300 mM acetate buffer (3.1 g CH_3_COONa and 16 ml CH_3_COOH), pH 3.6, 10 mM TPTZ (2, 4, 6-tripyridyl-*s*-triazine) solution in 40 mM HCl and 20 mM FeCl_3_ · 6H_2_O solution. The fresh working solution was prepared by mixing 25 ml acetate buffer, 2.5 ml TPTZ and 2.5 ml FeCl_3_ · 6H_2_O. Plant extracts (15 μl) were allowed to react with 285 μl of the FRAP solution for 30 min in dark. Observations of the colored product (ferrous tripyridyltriazine complex) were taken at 593 nm. The calibration curve was prepared from the equation y = 0.097x - 0.048, R^2^ = 0.993, where x was absorbance and y was FeSO_4_ concentration (mol). Linearity was achieved between 1 × 10^-4^ and 1 × 10^-3^ mol FeSO_4_. Results were expressed in mol Fe (II)/g of extract and compared with that of QR, AA and BHT.

### Total polyphenol assay

Total polyphenol content was measured using Folin-Ciocalteu colorimetric method as described by previous investigators [42]. The phytococktail methanol and n-hexane extracts (10 μl) was mixed with 20 μl of Folin-Ciocalteu reagent and 200 μl of H_2_O, and incubated at room temperature for 3 min. Following the addition of 100 μl of 20% sodium carbonate to the mixture, total polyphenol was determined after 1 h of incubation at room temperature. The absorbance of the resulting blue color was measured at 765 nm. Quantification was done with respect to the standard curve of gallic acid. The polyphenol content was expressed as gallic acid equivalent (GAE) using the following equation based on the calibration curve: y = 0.005x + 0.059, R^2^ = 0.987, where x was absorbance and y was GAE (mol/g of extract) at a final concentration of 100 μg/ml.

### Total flavonoid assay

Estimation of total flavonoid in the methanol and n-hexane extracts was carried out using the previous method [43]. Briefly, to 100 μl of sample, 100 μl of 2% AlCl_3_ ethanol solution was added. The contents were incubated for 1 h at room temperature and the absorbance was measured at 420 nm. Total flavonoid content was calculated as quercetin equivalent (QE) using the following equation based on the calibration curve: y = 0.011x + 0.038, R^2^ = 0.984, where x was absorbance and y was QE (mol/g of extract) at a final concentration of 100 μg/ml.

### Total flavonol assay

Total flavonol in the extracts was also estimated by the method described previously [44]. To 100 μl extract, 100 μl of 2% AlCl_3_ ethanol and 150 μl (50 g/l) sodium acetate solutions were added. The absorbance at 440 nm was measured after 2.5 h at 20°C. Total flavonol content was calculated as quercetin equivalent (QE) using the following equation based on the calibration curve: y = 0.016x + 0.001, R^2^ = 0.985, where x was absorbance and y was QE (mol/g of extract) at a final concentration of 100 μg/ml.

### Total proanthocyanidin assay

Total proanthocyanidin test was performed by vanillin-HCl assay with minor modification [45]. Vanillin reagent (1%) was prepared in methanol and incubated at 30°C before use. The working reagent was prepared by mixing one part of 1% vanillin solution and one part of 8% HCl solution in methanol. The reaction mixture contained working vanillin reagent (100 μl) and plant extracts (20 μl). The absorbance at 500 nm was measured after 20 min at 30°C. Total proanthocynidin content was calculated as catechin equivalent (CE) using the following equation based on the calibration curve: y = 0.327x + 0.039, R^2^ = 0.973, where x was absorbance and y was CE (mol/g of extract) at a final concentration of 100 μg/ml.

### Determination of total carotenoids

The phytococktail extracts of the appropriate concentration (1 mg/ml) were analyzed in spectrophotometer at 470, 653 and 666 nm. The concentration of total carotenoid was determined [46]. The carotenoid concentration was expressed in mg/g of extract.

### GC/MS analysis

#### Preparation of sample for GC/MS analysis

The 100 mg and 50 mg concentrated methanol and n-hexane extracts of phytococktail were dissolved in 25 ml of respective solvents, vortexed properly and filtered through 0.22 μm syringe filter (Millipore Corp., Bedford, MA, USA). One microlitre aliquot of the sample solution was then injected into the GC/MS MS system for the requisite analysis.

#### Instrumentation and chromatographic conditions

GC/MS analysis was carried out on a Thermo Finnigan PolarisQ Ion Trap GC/MS MS system comprising of an AS2000 liquid autosampler (Thermo Finnigan, Thermo Electron Corporation, Austin, TX, USA) and the peaks in the chromatogram were identified on the basis of their mass spectra as per our previous report [19]. The gas chromatograph was interfaced to a mass spectrometer instrument employing the following conditions *viz.* Durabond DB-5 ms column (30 m × 0.25 mm × 0.25 μm), operating in electron impact [electron ionisation positive (EI^+^)] mode at 70 eV, helium (99.999%) as carrier gas at a constant flow of 1 ml/min and an injection volume of 0.5 EI (split ratio of 10:1), injector temperature 280°C and transfer line temperature 300°C. The oven temperature was programmed from 50°C (isothermal for 2 min), with gradual increase in steps of 10°C/min, to 300°C. Mass spectra were taken at 70 eV, a scan interval of 0.5 s and full mass scan range from 25 m/z to 1000 m/z. The data acquisition was performed on Finnigan Xcalibur data acquisition and processing software version 2.0 (ThermoQuest, LC and LC/MS Division, San Jose, California, USA).

#### Identification of components

Interpretation of mass spectrum of GC/MS was done using the NIST/EPA/NIH Mass Spectral Database (NIST11), with NIST MS search program v.2.0 g [National Institute Standard and Technology (NIST), Scientific Instrument services, Inc., NJ, USA]. The mass spectrum of the unknown component was compared with the spectrum of the known components stored in the NIST library. The name, molecular weight and structure of the components of the test materials were ascertained.

### Statistical analysis

All the experimental results are expressed as mean ± standard deviation (SD) using statistical analysis with SPSS 17.0 (Statistical Program for Social Sciences, SPSS Corporation, Chicago, IL) version. Analysis of variance (ANOVA) in a completely randomized design, Duncan’s multiple range test and Pearson’s correlation coefficients were performed to compare the data. Post hoc analysis was performed using Neuman Keuls Test, and values with *p* < 0.05 were considered significant.

## Results

### DPPH radical scavenging capacity

The free radical scavenging capacity of the phytococktail methanol and n-hexane extracts and the three positive controls *viz.* QR, AA and BHT were compared through their ability to scavenge DPPH radical. The RSa_50_ values were found to be 393.57, 319, 8.09, 9.23 and 51.92 μg/ml for methanol extract, n-hexane extract, QR, AA and BHT respectively. The DPPH scavenging capacity of the phytococktail extracts and the positive controls, expressed as EC_50_ value, were 7.39, 5.99, 0.15, 0.17 and 0.98 μg/ml. The ARP values of methanol and n-hexane extracts were also calculated and were found to be 13.53 and 16.69 respectively. AEAC values of the two extracts were correspondingly 2345.19 and 2893.41 (Table 1).

**Table 1:** **Effect of phytococktail methanol and n-hexane extracts on DPPH and ABTS radical-scavenging capacities**^**a**^

**Concentration (μg/ml)**			**Scavenging capacity (%)**							
			**DPPH radical-scavenging capacity**			**ABTS radical-scavenging capacity**				
	**PCM**^**b**^	**PCH**^**c**^	**QR**^**d**^	**AA**^**e**^	**BHT**^**f**^	**PCM**	**PCH**	**QR**	**AA**	**BHT**
2.5	-	-	19.92 ± 1.53	17.71 ± 3.03	6.64 ± 0.78^#Φ^	-	-	35.24 ± 3.43	34.29 ± 1.75	7.43 ± 1.33^#Φ^
5	-	-	41.85 ± 5.12	32.23 ± 8.30	9.77 ± 2.95^#Φ^	-	-	64.76 ± 7.07	66.14 ± 15.94	13.91 ± 2.71^#Φ^
10	-	-	63.22 ± 4.50	63.93 ± 1.59	15.30 ± 1.13^#Φ^	-	-	86.84 ± 1.57	87.15 ± 0.92	27.71 ± 1.10^#Φ^
15	-	-	83.71 ± 8.59	77.93 ± 4.40	18.49 ± 0.56^#Φ^	-	-	87.79 ± 0.18	87.69 ± 0.18	34.39 ± 2.78^#Φ^
20	3.45 ± 2.26	31.45 ± 3.77^*^	87.83 ± 0.24^*$^	88.48 ± 0.90^*$^	25.33 ± 0.81^*$#Φ^	3.29 ± 0.49	3.40 ± 1.29	87.79 ± 0.18^*$^	88.00 ± 0.18^*$^	42.36 ± 0.55^*$#Φ^
40	10.48 ± 1.18	34.05 ± 3.10^*^	88.61 ± 0.24^*$^	88.93 ± 0.45^*$^	36.78 ± 5.88^*#Φ^	9.87 ± 1.39	4.25 ± 0.66^*^	87.79 ± 0.18^*$^	87.90 ± 0.00^*$^	60.40 ± 4.94^*$#Φ^
60	13.61 ± 0.30	35.29 ± 6.13^*^	88.93 ± 0.09^*$^	89.06 ± 0.39^*$^	58.07 ± 4.29^*$#Φ^	15.29 ± 3.23	8.81 ± 0.80^*^	88.11 ± 0.18 ^*$^	88.22 ± 0.55^*$^	74.84 ± 1.39^*$#Φ^
80	16.41 ± 0.34	41.15 ± 3.28^*^	88.93 ± 0.18^*$^	89.26 ± 0.39^*$^	62.89 ± 3.20^*$#Φ^	22.08 ± 0.18	22.93 ± 2.87	88.32 ± 0.37^*$^	88.22 ± 0.55^*$^	76.86 ± 0.49^*$#Φ^
100	20.38 ± 2.79	42.51 ± 1.44^*^	89.13 ± 0.24^*$^	89.58 ± 0.11^*$^	65.30 ± 3.07^*$#Φ^	30.15 ± 1.51	33.33 ± 3.80	88.43 ± 0.49^*$^	88.54 ± 0.32^*$^	81.00 ± 1.02^*$#Φ^
150	27.99 ± 1.59	44.14 ± 0.39^*^	-	-	-	44.27 ± 1.39	48.83 ± 2.12^*^	-	-	-
200	32.03 ± 2.07	44.66 ± 0.63^*^	-	-	-	57.22 ± 1.29	61.36 ± 2.41	-	-	-
250	34.18 ± 1.56	46.55 ± 4.06^*^	-	-	-	64.65 ± 1.65	68.90 ± 1.75^*^	-	-	-
300	39.78 ± 1.64	49.35 ± 0.49^*^	-	-	-	74.95 ± 1.29	73.57 ± 2.23	-	-	-
400	49.35 ± 2.47	52.80 ± 4.26	-	-	-	85.46 ± 0.80	75.58 ± 2.71^*^	-	-	-
500	60.81 ± 4.01	59.05 ± 5.21	-	-	-	87.79 ± 0.18	76.43 ± 3.91^*^	-	-	-
RSa_50_ values	393.57	319.00	8.09	9.23	51.92	181.98	183.37	3.42	3.39	33.15
EC_50_ values	7.39	5.99	0.15	0.17	0.98	2.84	2.86	0.05	0.05	0.52
ARP values	13.53	16.69	666.67	588.24	102.04	35.21	34.97	1926.72	1941.75	192.31
AEAC values	2345.19	2893.41				2170.56	2154.11			

^a^Mean ± SD of three replicates. *p* < 0.05: ^*^compared with PCM; ^$^compared with PCH; ^#^compared with quercetin; ^Φ^compared with ascorbic acid; ^b^Phytococktail methanol extract; ^c^Phytococktail n-hexane extract; ^d^Quercetin; ^e^Ascorbic acid; ^f^Butylated hydroxytoluene.

### ABTS radical scavenging capacity

The ABTS radical scavenging capacity (%) of methanol and n-hexane extracts of the phytococktail compared to QR, AA and BHT has been depicted in Table 1 and the scavenging capacity of the extracts on ABTS radical was similar to the results of the scavenging capacity on DPPH radical. The extracts scavenged the ABTS radical in a dose dependent manner at concentration of 20-500 μg/ml. The positive controls *viz*. QR, AA and BHT at concentration of 2.5-100 μg/ml were also found to produce dose dependent inhibition of ABTS radical. The quantity of methanol and n-hexane extracts required to produce 50% scavenging (RSa_50_) of ABTS radical were found to be 181.98 and 183.37 μg/ml respectively. Analogous effects were produced by QR, AA and BHT at concentration of 3.42, 3.39 and 33.15 μg/ml respectively. The EC_50_ and ARP values of the extracts were also comparable to the standards. EC_50_ values of methanol and n-hexane extracts were found to be 2.84 and 2.86 μg/ml respectively. QR, AA and BHT were set up at EC_50_ of 0.05, 0.05 and 0.52, respectively. The ARP values of the methanol and n-hexane extracts were 35.21 and 34.97, respectively. AEAC values of methanol and n-hexane extracts were found to be 2170.56 and 2154.11, respectively (Table 1).

### Inhibition of lipid peroxidation

Using egg yolk homogenates as lipid-rich medium of peroxidation, percentage lipid peroxidation inhibition by the phytococktail extracts and the positive controls *viz.* α-tocopherol, BHA and BHT has been shown in Table 2. The IC_50_ values for methanol, n*-*hexane, α-tocopherol BHA and BHT were 415.54, 29.53, 9.09, 5.57 and 27.01 μg/ml, respectively. Lipid peroxidation inhibitory capacity of the phytococktail extracts was found to increase with increasing concentration and was comparable to the positive standards. The n-hexane extract showed significantly higher inhibition capacity (*p* < 0.05) than the methanol extract. BHA was observed to have significantly higher inhibitory capacity (*p* < 0.05) compared with the methanol, n-hexane extract and the other two standards.

**Table 2:** **Effect of phytococktail methanol and n-hexane extracts on lipid peroxide and nitric oxide radical-scavenging capacities**^**a**^

**Concentration (μg/ml)**	**Inhibition (%) / Scavenging capacity (%)**							
			**Lipid peroxidation inhibition capacity**			**Nitric oxide radical-scavenging capacity**		
	**PCM**^**b**^	**PCH**^**c**^	**α-tocopherol**	**BHA**^**d**^	**BHT**^**e**^	**PCM**	**PCH**	**BHA**
2.5	21.92 ± 0.70	29.35 ± 1.15^*^	32.19 ± 2.10^*^	32.13 ± 2.17^*^	24.18 ± 0.70^$#Φ^	-	-	10.29 ± 0.87
5	25.11 ± 0.23	36.65 ± 0.42^*^	38.32 ± 2.13^*^	54.62 ± 3.59^*$#^	30.26 ± 1.83^*$#Φ^	-	-	14.88 ± 0.29
10	27.82 ± 0.50	39.14 ± 1.01^*^	56.61 ± 3.86^*$^	68.80 ± 6.12^*$#^	32.80 ± 4.69^#Φ^	-	-	18.74 ± 0.55
15	29.55 ± 0.31	41.97 ± 0.12^*^	62.98 ± 0.38^*$^	75.22 ± 1.00^*$#^	44.38 ± 0.48^*$#Φ^	-	-	21.89 ± 0.23
20	30.07 ± 0.46	44.13 ± 2.48^*^	66.19 ± 0.55^*$^	81.34 ± 1.09^*$#^	48.86 ± 0.64^*$#Φ^	1.18 ± 0.43	1.18 ± 0.43	25.54 ± 0.37^*$^
40	32.38 ± 0.65	46.92 ± 0.48^*^	70.73 ± 2.05^*$^	81.82 ± 0.71^*$#^	57.46 ± 7.40^*$#Φ^	3.58 ± 0.49	2.45 ± 0.42^*^	29.10 ± 0.66^*$^
50	32.38 ± 0.65	48.25 ± 0.10^*^	75.28 ± 0.99^*$^	82.99 ± 0.30^*$#^	67.42 ± 1.97^*$#Φ^	5.84 ± 0.43	3.15 ± 0.49	39.77 ± 2.67^*$^
60	39.22 ± 0.10	50.50 ± 0.42^*^	79.10 ± 0.55^*$^	87.34 ± 0.90^*$#^	79.16 ± 3.10^*$Φ^	8.10 ± 0.65	4.57 ± 0.43^*^	42.38 ± 0.98^*$^
80	42.38 ± 0.37	54.49 ± 0.56^*^	81.22 ± 0.46^*$^	88.12 ± 1.34^*$#^	79.70 ± 1.47^*$Φ^	11.36 ± 0.43	5.84 ± 0.86^*^	44.89 ± 0.36^*$^
100	43.41 ± 0.11	57.16 ± 0.45^*^	85.46 ± 2.28^*$^	89.33 ± 89.33^*$#^	80.18 ± 1.14^*$#Φ^	13.19 ± 0.25	7.12 ± 0.43^*^	48.12 ± 1.49^*$^
150	44.56 ± 0.37	60.61 ± 0.28^*^	85.74 ± 0.19^*$^	89.78 ± 0.21^*$#^	80.75 ± 0.20^*$#Φ^	14.90 ± 0.65	8.53 ± 0.24^*^	50.58 ± 0.91^*$^
200	45.53 ± 0.28	62.98 ± 0.28^*^	86.57 ± 0.31^*$^	90.24 ± 0.16^*$#^	81.58 ± 0.90^*$#Φ^	18.15 ± 0.73	9.38 ± 0.49^*^	53.21 ± 0.65^*$^
250	46.98 ± 0.46	64.80 ± 0.21^*^	86.81 ± 0.10^*$^	90.57 ± 0.10^*$#^	82.29 ± 0.36^*$#Φ^	20.83 ± 1.07	9.94 ± 0.24^*^	53.49 ± 0.14^*$^
300	48.19 ± 0.32	66.91 ± 0.48^*^	87.11 ± 0.35^*$^	90.64 ± 0.15^*$#^	84.02 ± 0.50^*$#Φ^	23.23 ± 0.43	10.93 ± 0.85^*^	53.85 ± 0.15^*$^
400	50.74 ± 0.18	68.43 ± 0.21^*^	87.56 ± 0.33^*$^	91.09 ± 0.70^*$#^	85.76 ± 0.42^*$#Φ^	25.35 ± 0.43	12.21 ± 0.43^*^	54.19 ± 0.43^*$^
500	50.74 ± 0.18	69.58 ± 0.27^*^	90.23 ± 0.66^*$^	92.33 ± 0.38^*$#^	88.61 ± 1.94^*$Φ^	29.03 ± 0.88	13.05 ± 0.43^*^	56.31 ± 0.43^*$^
IC_50_ / RSa_50_ values	415.54	29.53	9.09	5.57	27.01	828.87	2009.21	148.69

^a^Mean ± SD of three replicates. *p* < 0.05: ^*^compared with PCM; ^$^compared with PCH; ^#^compared with α-tocopherol; ^Φ^compared with BHA; ^b^Phytococktail methanol extract; ^c^Phytococktail n-hexane extract; ^d^Bulylated hydroxyanisole; ^e^Butylated hydroxytoluene.

### Nitric oxide radical scavenging capacity

The scavenging capacity of the phytococktail extracts against nitric oxide released by sodium nitroprusside was studied and the result has been depicted in Table 2. The percentage radical scavenging capacity of the extracts and the reference standard BHA against nitric oxide radical was increased in a dose dependent mode. The RSa_50_ values for methanol, n*-*hexane and BHA were 828.87, 2009.21 and 148.69 μg/ml, respectively. The methanol extract showed significantly higher radical scavenging capacity (*p* < 0.05) in comparison with the n*-*hexane extract.

### Total antioxidant power (FRAP)

The ability of the plant extracts to reduce ferric ions was determined using the FRAP assay [41]. An antioxidant capable of donating a single electron to the ferric-TPTZ (Fe(III)-TPTZ) complex would cause the reduction of this complex into the blue ferrous-TPTZ (Fe(II)-TPTZ) complex which absorbs strongly at 593 nm. The FRAP values were found to be 8.21306 × 10^-4^ and 1.03436 × 10^-3^ mol Fe (II)/g of methanol and n-hexane extract respectively. The FRAP values for the phytococktail extracts were significantly lower than that of QR, AA and BHT (Additional file 1: Table S1).

### Total polyphenol, flavonoid, flavonol, proanthocyanidin and carotenoid content

In the present study, the content of total polyphenol in methanol and n-hexane extracts of the phytococktail was found to be 2.3416 × 10^-4^ and 2.899 × 10^-4^ mol/g of extract as expressed in gallic acid equivalent. The concentration of flavonoid in the two extracts was found to be 4.21 × 10^-5^ and 6.11 × 10^-5^ mol quercetin/g of extract. The content of flavonol in the phytococktail extracts was 1.068 × 10^- 4^ and 9.85 × 10^-5^ mol/g of extract as expressed in quercetin equivalent. Total proanthocyanidin content in the phytococktail extracts was measured to be 3 × 10^-7^ and 2 × 10^-7^ mol catechin/g of extract. Quantity of carotenoid in the two extracts was 3.5004 × 10^-1^ and 4.87395 × 10^-2^ mg/g of extract (Additional file 1: Table S2).

### GC/MS chemometric profile of the phytococktail extracts

GC/MS chromatograms of n-hexane and methanol extracts of the phytococktail as per the aforementioned experimental procedure showed various peaks indicating the presence of different chemotypes in the respective extracts.

### Methanol extract

**Figure 1:**
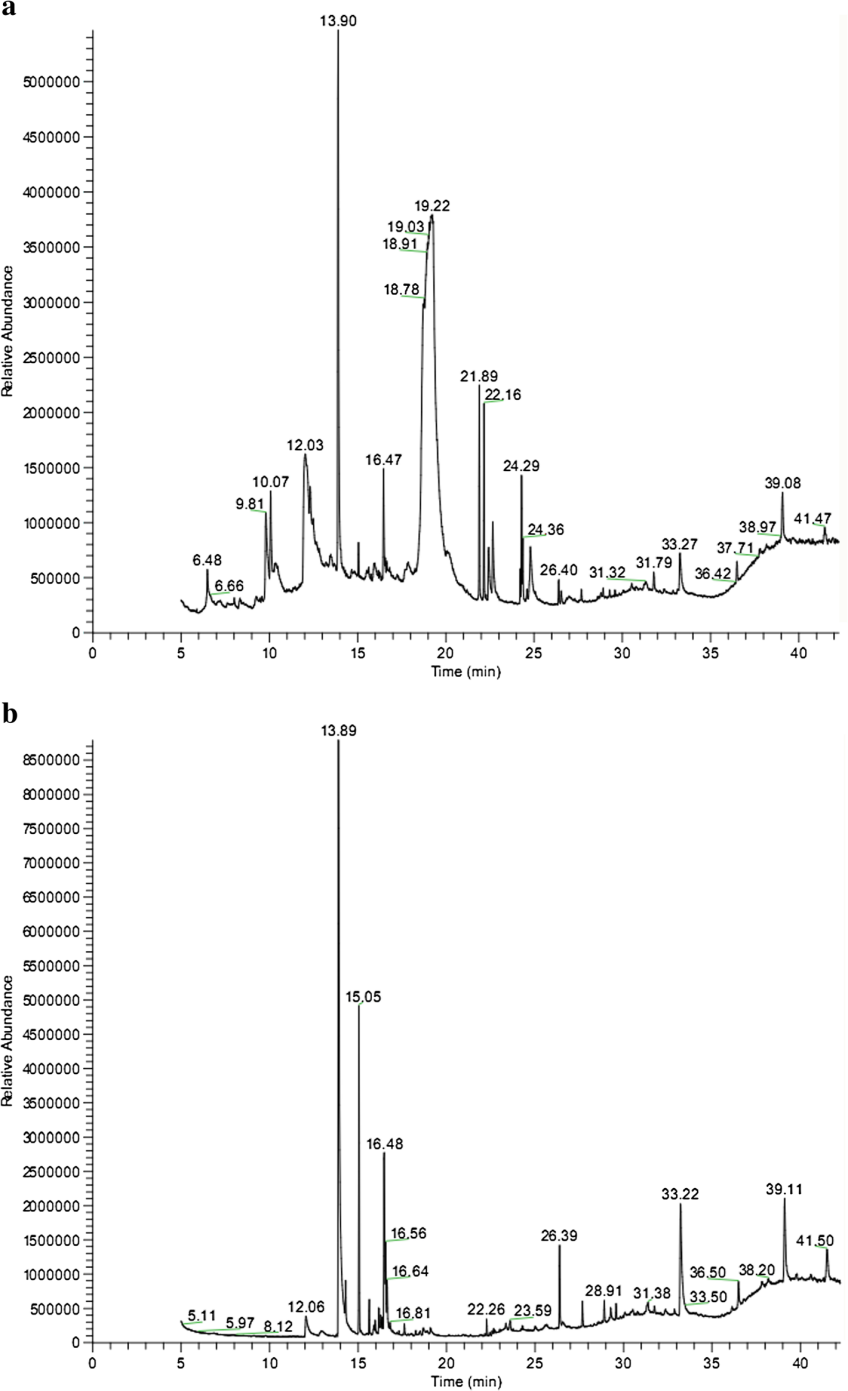
GC/MS chromatogram of phytococktail a. methanol extract, b. n-hexane extract.

The methanol extract revealed the presence of 19 different chemotypes which were characterized and identified by comparison of their mass fragmentation patterns with those in the NIST database library (Table 3, Figure 1a). Among these 19 chemotypes, α-D-glucopyranoside, O-α-D-glucopyranosyl-(1.fwdarw.3)-β-D-fructofuranosyl (53.35%), 2-furancarboxaldehyde, 5-(hydroxymethyl) (13.17%), eugenol (7.26%) and τ-sitosterol (4.31%) were found to be major constituents whereas, malic acid, dimethyl ester (2.72%), oleic acid (2.48%), 2-methoxynaphthalene (2.46%), aceteugenol (2.07%), methyl oleate (1.82%), 3-hydroxypyridine-N-oxide (1.67%), palmitic acid (1.66%), methyl palmitoleate (1.66%), methyl palmitate (1.43%), piperine (1.24%), palmitoleic acid (1.07%), 1-methyl-4-hydroxybenzoate (0.81%), stigmastanol (0.71%), *trans*-caryophyllene (0.29%) and α-tocopherol (0.22%) were found to be present in trace amount.

**Table 3:** Chemometric profile of methanol extract of phytococktail

**S. No.**	**Peak RT (min)**	**Peak area**	**Peak area (%)**	**Compound detected**	**Major group**	**Hit**	**SI**	**RSI**	**Prob**	**CAS No**	**Mol. formula**	**Mol. Wt.**	**Content (mg/g)**
1	6.48	4314646	1.67	3-Hydroxypyridine-N-oxide	Alkaloid derivative	1	664	887	46.53	6602-28-4	C_5_H_5_NO_2_	111	0.0501
2	9.81	7021309	2.72	Malic acid, dimethyl ester	Organic acid ester	1	717	821	22.44	1587-15-1	C_6_H_10_O_5_	162	0.0816
3	10.07	6345493	2.46	2-Methoxynaphthalene	Aromatic ether	1	702	709	50.19	93-04-9	C_11_H_10_O	158	0.0738
4	12.03	33982819	13.17	2-Furancarboxaldehyde, 5-(hydroxymethyl)	Aldehyde	1	735	800	85.35	67-47-0	C_6_H_6_O_3_	126	0.3951
5	13.90	18735029	7.26	Eugenol	Phenylpropanoid	1	861	884	29.07	97-53-0	C_10_H_12_O_2_	164	0.2178
6	15.05	731414	0.29	*trans-*Caryophyllene	Terpenoid	1	742	829	4.78	87-44-5	C_15_H_24_	204	0.0087
7	15.97	2081450	0.81	1-Methyl-4-hydroxybenzoate	Organic acid ester	1	636	790	18.25	99-76-3	C_8_H_8_O_3_	152	0.0243
8	16.47	5342706	2.07	Aceteugenol acetate	Phenylpropanoid derivative	3	836	852	18.05	93-28-7	C_12_H_14_O_3_	206	0.0621
9	19.21	137973198	53.35	α-D-glucopyranoside, O-α-D-glucopyranosyl-(1.fwdarw.3)-β-D-fructofuranosyl	Glycoside	1	696	704	16.21	597-12-6	C_18_H_32_O_16_	504	1.6005
10	21.89	4275714	1.66	Methyl palmitoleate	Fatty acid ester	1	837	843	54.57	1120-25-8	C_17_H_32_O_2_	268	0.0498
11	22.16	3692581	1.43	Methyl palmitate	Fatty acid ester	1	769	807	66.81	112-39-0	C_17_H_34_O_2_	270	0.0429
12	22.42	2765978	1.07	Palmitoleic acid	Fatty acid	1	783	800	58.89	2091-29-4	C_16_H_30_O_2_	254	0.0321
13	22.66	4283613	1.66	Palmitic acid	Fatty acid	1	765	800	58.65	57-10-3	C_16_H_32_O_2_	256	0.0498
14	24.29	4700456	1.82	Methyl oleate	Fatty acid ester	1	779	809	15.15	112-62-9	C_19_H_36_O_2_	296	0.0546
15	24.79	6407869	2.48	Oleic acid	Fatty acid	1	758	775	20.74	112-80-1	C_18_H_34_O_2_	282	0.0744
16	33.27	3211489	1.24	Piperine	Alkaloid	1	603	733	69.74	94-62-2	C_17_H_19_NO_3_	285	0.0372
17	36.49	558710	0.22	α-Tocopherol	Phytosterol	1	649	715	64.90	59-02-9	C_29_H_50_O_2_	430	0.0066
18	39.10	11115657	4.31	τ-Sitosterol	Phytosterol	2	691	762	34.65	83-47-6	C_29_H_50_O	414	0.1293
19	41.47	794822	0.31	Unknown	-	-	-	-	-	-	-	-	0.0093

### n-Hexane extract

GC/MS chemometric profile of the phytococktail n-hexane extract showed the presence of 21 different chemotypes (Table 4, Figure 1b). Among these 21 chemotypes, eugenol (41.93%), piperine (10.87%), aceteugenol (8.28%), *trans*-caryophyllene (8.41%) and τ-sitosterol (6.62%) were found to be present in major amount whereas, δ-cadinene (3.08%), isoledene (2.55%), cuminic aldehyde (2.46%), calamenene (2.44%), α-tocopherol (1.55%), α-levantenolide (1%), α-humulene (0.96%), cedr-8-ene (0.91%), α-muurolene (0.81%), bis(2-ethylhexyl)phthalate (0.69%), 3-methoxy-5-methylphenol (0.43%), ar-curcumene (0.61%), α-amorphene (0.40%), caryophyllene oxide (0.36) and 1,3-bis(cinnamoyloxymethyl)adamantane (0.29%) were found to be present in trace amount.

**Table 4:** Chemometric profile of n-hexane extract of phytococktail

**S. No.**	**Peak RT (min)**	**Peak area**	**Peak area (%)**	**Compound detected**	**Major group**	**Hit**	**SI**	**RSI**	**Prob**	**CAS No**	**Mol. formula**	**Mol. Wt.**	**Content (mg/g)**
1	12.06	2819064	2.46	Cuminic aldehyde	Aromatic aldehyde	3	793	856	22.00	122-03-2	C_10_H_12_O	148	0.0246
2	12.92	335612	0.29	1,3-Bis(cinnamoyloxymethyl) adamantane	Ether	2	712	723	19.29	303797-58-2	C_30_H_32_O_4_	456	0.0029
3	13.89	48606490	41.93	Eugenol	Phenylpropanoid	1	877	879	18.05	97-53-0	C_10_H_12_O_2_	164	0.4193
4	14.29	2923628	2.55	Isoledene	Terpenoid	1	707	721	14.90	95910-36-4	C_15_H_24_	204	0.0255
5	15.05	9616594	8.41	*trans*-Caryophyllene	Terpenoid	1	862	866	13.91	87-44-5	C_15_H_24_	204	0.0841
6	15.63	1104181	0.96	α-Humulene	Terpenoid	1	821	837	9.62	6753-98-6	C_15_H_24_	204	0.0096
7	15.90	458653	0.40	α-Amorphene	Terpenoid	1	763	841	5.04	23515-88-0	C_15_H_24_	204	0.004
8	15.97	697731	0.61	ar-Curcumene	Terpenoid	1	785	801	71.73	644-30-4	C_15_H_22_	202	0.0061
9	16.18	1038187	0.91	Cedr-8-ene	Terpenoid	1	799	805	9.62	469-61-4	C_15_H_24_	204	0.0091
10	16.27	927957	0.81	α-Muurolene	Terpenoid	1	821	850	7.06	31983-22-9	C_15_H_24_	204	0.0081
11	16.48	9478971	8.28	Aceteugenol	Phenylpropanoid	1	864	873	19.60	93-28-7	C_12_H_14_O_2_	206	0.0828
12	16.56	3528046	3.08	δ-Cadinene	Terpenoid	1	760	853	19.23	483-76-1	C_15_H_24_	204	0.0308
13	16.64	2791591	2.44	Calamenene	Terpenoid	6	642	699	8.58	483-77-2	C_15_H_22_	202	0.0244
14	17.62	413127	0.36	Caryophyllene oxide	Terpenoid	1	789	812	39.62	1139-30-6	C_15_H_24_O	220	0.0036
15	26.39	3168547	2.77	Unknown	-	-	-	-	-	-	-	-	0.0277
16	27.68	1134640	1.00	α-Levantenolide	Terpenoid	1	620	728	55.67	30987-48-5	C_20_H_30_O_3_	318	0.01
17	28.91	791295	0.69	Bis(2-ethylhexyl)phthalate	Ester	1	685	741	13.24	117-81-7	C_24_H_38_O_4_	390	0.0069
18	33.22	12440863	10.87	Piperine	Alkaloid	1	753	815	86.19	94-62-2	C_17_H_19_NO_3_	285	0.1087
19	36.50	1764098	1.55	α-Tocopherol	Phytosterol	1	683	728	39.19	59-02-9	C_29_H_50_O_2_	430	0.0155
20	39.11	7572383	6.62	τ-Sitosterol	Phytosterol	1	768	807	64.55	83-47-6	C_29_H_50_O	414	0.0662
21	41.50	3433999	3.01	Unknown	-	-	-	-	-	-	-	-	0.0301

## Discussion

Progression of a large number of common chronic diseases is induced by free radical-mediated oxidative damage and a lot of health benefits are attributed to the utilization of fruits and vegetables in our diet due to their strong antioxidant capacities. A wide variety of biologically active phytochemicals such as polyphenols, flavonoids, alkaloids, terpenoids, carotenoids etc. are derived from plant foods and natural products which have promising health benefits. These diverse phytocompounds have protective effects against chronic diseases while acting in combination rather than individually [47]. In recent times, the antioxidant content has become an essential biochemical marker of plant product quality. The antioxidant capacity resulting from hydrophilic or lipophilic compounds individually has been estimated in plant foods. In the present work, we have performed the widely used and well recognized antioxidant capacity assays that have positive influence and applications in biological antioxidant research. The DPPH radical scavenging assay is a simple and precise method to measure the antioxidant capacity of plant extracts where the DPPH radical is used as a stable free radical to determine the antioxidant capacity of natural compounds. In our study, the DPPH radical scavenging capacity of the phytococktail extracts was found to increase in a dose dependent manner. The phytococktail extracts at the used concentrations displayed potential free radicals scavenging effect (Table 1). A higher DPPH radical scavenging capacity is associated with a lower RSa_50_ value. The DPPH radical is considered as a model for lipophilic radical and from our result, it is evident that the phytococktail n-hexane extract showed significantly higher (*p* < 0.05) inhibition of DPPH radical as compared to methanol extract at concentration of 20-300 μg/ml. This result may be due to the activity of higher amount of lipophilic antioxidants present in the n-hexane extract in comparison with methanol extract. The ABTS assay is of great relevance to the study of both hydrophilic and lipophilic antioxidants as well as pure compounds and food extracts [38]. The antioxidant capacity measured by ABTS assay was determined by the decolorization of ABTS^.+^, by measuring the reduction of radical cation as percentage inhibition of absorbance at 734 nm. The results of ABTS assay revealed the same phenomenon where the n-hexane extract showed significantly higher (*p* < 0.05) radical scavenging property than methanol extract at 20, 40, 150 and 250 μg/ml concentrations. For measuring the RSa_50_ values accurately, we took a long concentration range (from very low to very high concentration) of the test materials. In contrast to the more direct methods for measuring antioxidant capacities of plant extracts, the FRAP assay is derived from a different redox reaction and due to its low cost, rapidity and technical simplicity, it has become a valuable method for detecting total antioxidant/reducing power of plant extracts. It has also been proven to produce values that have positive correlation with the results achieved by direct antioxidant capacity assays for various phytofoods [48]. Both extracts of the phytococktail were found to have high FRAP values which signify their high antioxidant potential. Our results are in agreement with the previous findings, where antioxidant capacity assays using DPPH·, ABTS·^+^ and FRAP exhibited analogous results [49].

In oxidative stress condition, intracellular and membrane lipids lose a hydrogen atom from an unsaturated fatty acyl chain and initiate lipid peroxidation that propagates as a chain reaction to generate a diverse array of peroxides and cyclic endoperoxides that produce a pink chromogen on reaction with thiobarbituric acid with highest absorbance at 532 nm, thus provide an estimate of lipid peroxidation inhibition [50]. Lipid peroxidation leads to an elevated oxidative stress in cells and induces numerous pathophysiological processes for disease development. Hence, inhibition of lipid peroxidation is a crucial property of the antioxidants present in plant extract by which they can alleviate the oxidative stress induced diseases [51]. In our study, the n*-*hexane extract displayed high inhibitory capacity which may be due to the presence of lipophilic antioxidants in this extract. The methanol extract also exhibited lipid peroxidation inhibition property. Thus, it can be assumed that the methanol and n*-*hexane extracts of the phytococktail could be beneficial in preventing the oxidative damage and uphold the cellular, structural and functional integrity in stressful environments.

Nitric oxide plays vital role in the pathogenesis of several inflammatory diseases and other health problems [52]. In aqueous solution (with physiological pH) nitric oxide radical is generated from sodium nitroprusside and reacts with oxygen to form nitrogen oxide radicals which are scavenged by plant extracts through direct competition with oxygen and other oxides in the reaction medium [53]. In the present investigation, both n-hexane and methanol extracts of the phytococktail showed potential antioxidant capacity by scavenging the nitric oxide radicals. Therefore, the phytococktail could be useful in ameliorating a large number of diseases caused by inflammation and cellular damage.

**Figure 2:**
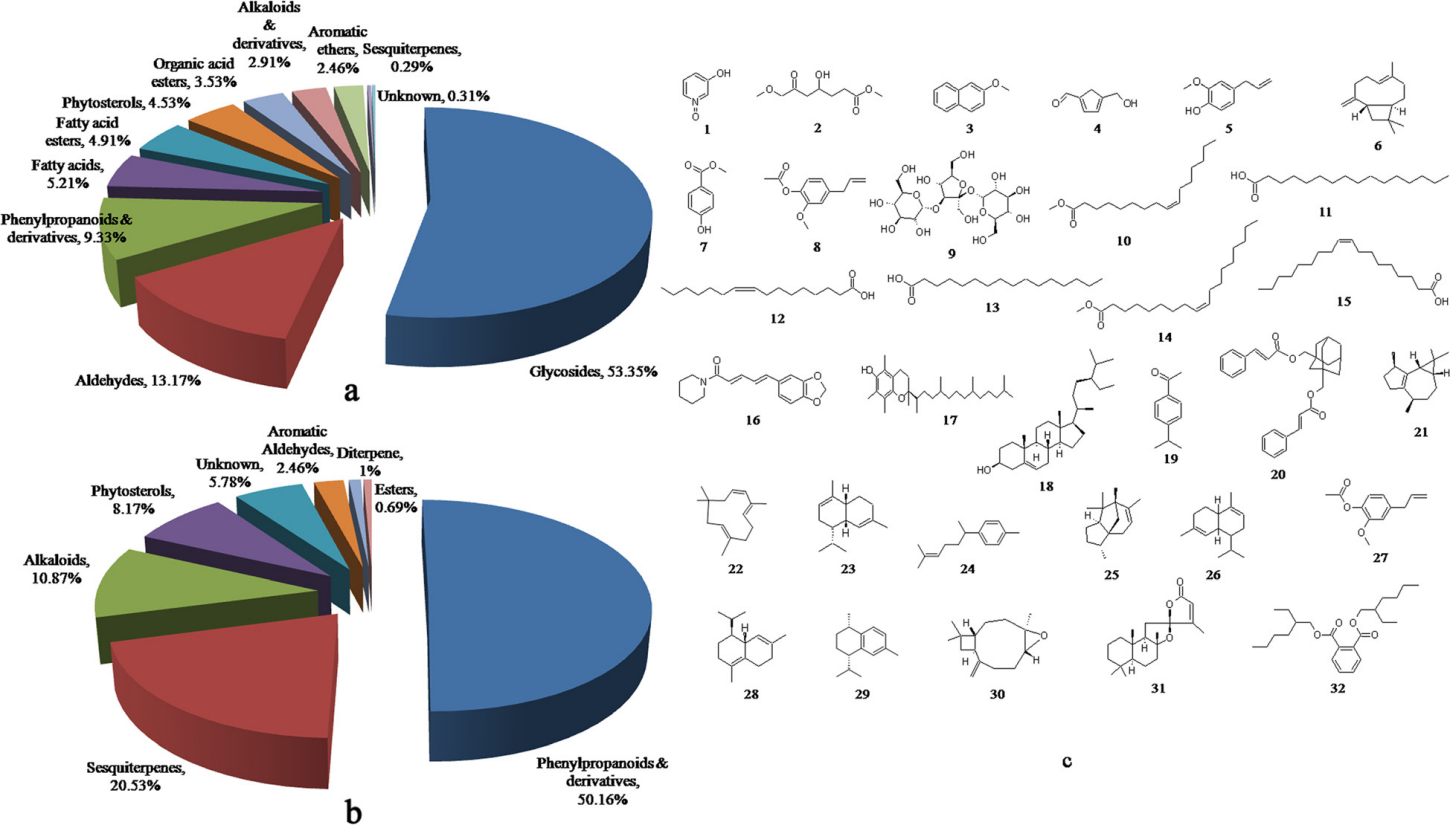
**Major phytochemical groups in phytococktail extracts. a**. methanol extract, **b**. n-hexane extract. **c**. phyto-chemotypes identified in methanol and n-hexane extracts of phytococktail.1: 3-Hydroxypyridine-N-oxide; 2: Malic acid, dimethyl ester; 3: 2-Methoxynaphthalene; 4: 2-Furancarboxaldehyde, 5-(hydroxymethyl); 5: Eugenol; 6: *trans-*Caryophyllene; 7: 1-Methyl-4-hydroxybenzoate; 8: Aceteugenol acetate; 9: α-D-glucopyranoside, O-α-D-glucopyranosyl-(1.fwdarw.3)-β-D-fructofuranosyl; 10: Methyl palmitoleate; 11: Methyl palmitate; 12: Palmitoleic acid; 13: Palmitic acid; 14: Methyl oleate; 15: Oleic acid; 16: Piperine; 17: α-Tocopherol; 18: τ-Sitosterol; 19: Cuminic aldehyde; 20: 1,3-Bis(cinnamoyloxymethyl) adamantane; 21: Isoledene; 22: α-Humulene; 23: α-Amorphene; 24: ar-Curcumene; 25: Cedr-8-ene; 26: α-Muurolene; 27: Aceteugenol; 28: δ-Cadinene; 29: Calamenene; 30: Caryophyllene oxide; 31: α-Levantenolide; 32: Bis(2-ethylhexyl)phthalate.

The phenolic compounds derived from plants are known to be powerful chain breaking natural antioxidants. The use of phenolics in the food industry is increasing because they retard oxidative degradation of lipids and thereby improve the quality and nutritional value of food. Flavonoids are natural phenolic compounds and well known antioxidants. In various studies, the plant extracts rich in flavonoids were found to have high antioxidant capacity. Flavonols are the major class of flavonoids present in a variety of fruits and vegetables and possess high antioxidant and antiradical capacity with many therapeutic applications [54,55]. Plant fruits contain carotenoids that also play an important role in human diet with their ability to act as free radical scavengers. The most widespread secondary metabolites in the plant kingdom reported so far are the phenolics and they have received great attention as potential natural antioxidant in terms of their ability to act as both efficient radical scavengers and metal chelator. Our results are in agreement with previous studies that showed significant positive correlation between total phenolic contents and antioxidant capacities of plant extracts [56]. From our results of phytochemical constituents present in the phytococktail (Additional file 1: Table S2, Figure 2), it is apparent that the antioxidant capacities of the phytococktail methanol extract can be attributed mainly to total polyphenol, flavonol, proanthocyanidin, glycoside, aldehyde and phenylpropanoid whereas, the antioxidant capacities of n-hexane extract can primarily be ascribed to total polyphenol, flavonoid, carotenoid, phenylpropanoid, terpenoid and alkaloid content. Therefore, the presence of these lipophilic and hydrophilic antioxidants identified during the phytochemical characterization could have contributed to the high antioxidant capacities of the extracts. The results of correlation analysis suggest that all the antioxidant capacity assays were significantly (*p* ≤ 0.05, *p* ≤ 0.01) associated with the total contents of different bioactive compounds in the phytococktail extracts (Additional file 1: Table S3). The berries of sea buckthorn have been well reported to contain a significant amount of natural antioxidants [17]. These were the prime raw ingredient of the phytococktail and may contribute to the total antioxidant capacity. In addition, apricot and roseroot also possess a diverse array of bioactive compounds [6,18-20] which could be responsible for the elevated antioxidant properties of the phytococktail extracts.

We have determined the bioactive volatile and semivolatile components in the phytococktail by GC/MS chemometric profiling. In medicinal chemistry it is very essential to ascertain the chemotyping of natural products that will allow us to scientifically determine and validate their traditional uses, pharmacological activities and therapeutic potential. Among the total compounds analysed by GC/MS, phenylpropanoids and their derivatives (59.49%) was the major cluster found in the n-hexane extract followed by sesquiterpenes (20.53%), alkaloids and derivatives (10.87%) and phytosterols (8.17%). In methanol extract glycosides (53.35%) was present in major amount followed by aldehydes (13.17%), phenylpropanoid derivatives (9.33%), fatty acids (5.21%), fatty acid esters (4.91%), phytostrerols (4.53%), organic acid esters (3.53%) and alkaloids and derivatives (2.91%). Relative abundance of major compounds in methanol and n-hexane extracts has been illustrated in Additional file 2: Figure S1 and Additional file 3: Figure S2. The order of extraction capacities of different polarity solvents for phenylpropanoids and derivatives, phytosterols, alkaloids and derivatives and sesquiterpenes was as follows: phenylpropanoids and derivatives: methanol (9.33%), n-hexane (50.16%); phytosterols: methanol (4.53%), n-hexane (8.17%); alkaloids and derivatives: methanol (2.91%), n-hexane (10.87%); sesquiterpenes: methanol (0.29%), n-hexane (20.53%) (Figure 2). All these compounds identified by GC/MS analysis were further investigated for their biological activities in Dr. Duke’s Phytochemical and Ethnobotanical Databases [57] which revealed that they possess a diverse range of positive pharmacological actions (Additional file 1: Table S4). Eventually, in the present study we have found glycosides, phenylpropanoids and derivatives, terpenoids, alkaloids, phytosterols, fatty acids and esters as the major groups of phyto-chemotypes in the extracts which are extremely beneficial for improving human health. These compounds have a broad range of pharmacological and therapeutic potential and could also be responsible for the high antioxidant capacities of the phytococktail*.*

In the present work it was established that the phytococktail extracts contained a considerable amount of diverse bioactive compounds with high antioxidant capacities. A significant and linear correlation was established between the antioxidant capacities and bioactive principles, demonstrating that these compounds could be the major contributors to antioxidant capacities. A total of 32 phyto-chemotypes have been identified from the methanol and n-hexane extracts of the phytococktail by GC/MS (Figure 2c). However, isolation of individual phyto-chemotypes and subjecting them to biological activity will definitely give fruitful results which may lead to the development of a novel drug.

## Conclusion

The phytococktail extracts contain various bioactive chemotypes having pharmaceutical importance and antioxidant properties. The phytococktail can definitely be used as an alternative source of natural antioxidants with consequential health promoting effects in the oxidative stress conditions. As a whole, it can be concluded that the phytococktail could be an aid in the stressful environment of high altitude as it conduce the maximum health benefit under a number of pattern of antioxidant capacity.

## Competing interests

The authors declare that they have no competing interests.

## Authors’ contributions

PD, PKB, ABT, OPC and SBS conceived and designed the experiments. PD, PKB and ABT performed the experiments and analyzed the data. PD, PKB and ABT contributed reagents/materials/analysis tools. PD and ABT wrote the manuscript. OPC, RBS and SBS did the concise review. All authors read and approved the final manuscript.

## Pre-publication history

The pre-publication history for this paper can be accessed here:

http://www.biomedcentral.com/1472-6882/13/259/prepub

## Supplementary Material

Additional file 1: Table S1 Ferric reducing antioxidant power (FRAP) of phytococktail methanol and n-hexane extracts ^a^. **Table S2.** Estimation of phytotochemical contents of phytococktail methanol and n-hexane extracts ^a^. **Table S3.** Pearson’s correlation coefficients between bioactive phyto-compounds and antioxidant capacity of PCM ^a^ and PCH ^b^. **Table S4.** Biological activities of active principles present in phytococktail extracts. Click here for file

Additional file 2: Figure S1 Relative abundance of major compounds in methanol extract. **a**. 2-Furancarboxaldehyde, 5-(hydroxymethyl)- , **b**. Eugenol, **c**. α-D-glucopyranoside, O-α-D-glucopyranosyl-(1.fwdarw.3)-β-D-fructofuranosyl, **d**. τ-Sitosterol. Click here for file

Additional file 3: Figure S2 Relative abundance of major compounds in n-hexane extract. **a**. Eugenol, **b**. *trans*-Caryophyllene, **c**. Aceteugenol, **d**. Piperine. Click here for file
