# Supplementary material for: Chemical composition and antioxidant capacities of phytococktail extracts from trans-Himalayan cold desert
Source: BMC Complement Altern Med. 2013 Oct 7;13:259. doi: 10.1186/1472-6882-13-259 (PMC3854071; doi:10.1186/1472-6882-13-259)
Supplement: Additional file 1: Table S1 — Ferric reducing antioxidant power (FRAP) of phytococktail methanol and n-hexane extracts a. Table S2. Estimation of phytotochemical contents of phytococktail methanol and n-hexane extracts a. Table S3. Pearson’s correlation coefficients between bioactive phyto-compounds and antioxidant capacity of PCM a and PCH b. Table S4. Biological activities of active principles present in phytococktail extracts. [file 1472-6882-13-259-S1.doc]

**Supplementary Table 1**

**Ferric reducing antioxidant power (FRAP) of phytococktail methanol and n-hexane extracts. ^a^**

| Extracts | FRAP (mol Fe (II)/g) |
| --- | --- |
| PCM ^b^ | 8.21306×10^-4^ ± 2.976×10^-5^ |
| PCH ^c^ | 1.03436×10^-3^ ± 5.952×10^-6^ |
| QR ^d^ | 1.02096×10^-3^ ± 1.61146×10^-4*$^ |
| AA ^e^ | 1.01993×10^-2^ ± 7.75415×10^-4*$^ |
| BHT ^f^ | 3.18213×10^-3^ ± 2.78605×10^-4*$#&^ |

^a^ Mean ± SD of three replicates.

^b^ Phytococktail methanol extract; ^c^ Phytococktail n-hexane extract; ^d^ Quercetin; ^e^ Ascorbic acid; ^f^ Butylated hydroxytoluene

*p* < 0.05: ^*^ compared with PCM; ^$^ compared with PCH; ^#^ compared with QR; ^&^ compared with AA

**Supplementary Table 2**

Estimation of phytotochemical contents of phytococktail methanol and n-hexane extracts. ^a^

| Phyto-components | PCM ^b^ | PCH ^c^ |
| --- | --- | --- |
| Total Polyphenol (mol gallic acid/g extract) | 2.899×10^-4^ ± 1.64×10^-5^ | 2.341×10^-4^ ± 1.79×10^-5^ |
| Total Flavonoid (mol quercetin/g extract) | 6.11×10^-5^ ± 3.29×10^-5^ | 4.21×10^-5^ ± 7.9×10^-6^ |
| Total Flavonol (mol quercetin/g extract) | 1.068×10^-4^ ± 3.1×10^-6^ | 9.85×10^-5^ ± 5.1×10^-6^ |
| Total Proanthocyanidin (mol catechin/g extract) | 3×10^-7^ ± 1×10^-7^ | 2×10^-7^ ± 1×10^-7^ |
| Total Carotenoid (mg/g extract) | 3.5004×10^-1^ ± 1.2632×10^-1^ | 4.87395×10^-2^ ± 9.616^*^ |

^a^ Mean ± SD of three replicates.

^b^ Phytococktail methanol extract; ^c^ Phytococktail n-hexane extract

*p* < 0.05: ^*^ compared with PCM

**Supplementary Table 3**

Pearson’s correlation coefficients between bioactive phyto-compounds and antioxidant capacity of PCM ^a^ and PCH ^b^.

| Bioactive phyto-compounds | PCM-ABTS ^c^ | PCH-ABTS ^d^ | PCM-DPPH ^e^ | PCH-DPPH ^f^ | PCM-LPI ^g^ | PCH-LPI ^h^ | PCM-NOS ^i^ | PCH-NOS ^j^ | PCM-FRAP ^k^ | PCH-FRAP ^l^ |
| --- | --- | --- | --- | --- | --- | --- | --- | --- | --- | --- |
| Total Polyphenol | 0.956^**^ | 0.857^**^ | 0.975^**^ | 0.815^**^ | 0.883^**^ | 0.857^**^ | 0.945^**^ | 0.884^**^ | 0.997^**^ | 0.942^**^ |
| Total Flavonoid | 0.548^**^ | 0.435^*^ | 0.603^**^ | 0.450^**^ | 0.551^**^ | 0.482^**^ | 0.576^**^ | 0.531^**^ | 0.621^**^ | 0.567^**^ |
| Total Flavonol | 0.960^**^ | 0.895^**^ | 0.979^**^ | 0.913^**^ | 0.888^**^ | 0.903^**^ | 0.949^**^ | 0.912^**^ | 0.998^**^ | 0.998^**^ |
| Total Proanthocyanidin | 0.764^**^ | 0.767^**^ | 0.737^**^ | 0.865^**^ | 0.712^**^ | 0.784^**^ | 0.764^**^ | 0.767^**^ | 0.779^**^ | 0.871^**^ |
| Total Carotenoid | 0.868^**^ | 0.899^**^ | 0.911^**^ | 0.901^**^ | 0.797^**^ | 0.905^**^ | 0.851^**^ | 0.919^**^ | 0.919^**^ | 0.999^**^ |

^*^ Signiﬁcant at *p* ≤ 0.05

^**^Signiﬁcant at *p* ≤ 0.01

^a^ PCM: Phytococktail methanol extract; ^b^ PCH: Phytococktail n-hexane extract

^c^ ABTS radical scavenging capacity of PCM; ^d^ ABTS radical scavenging capacity of PCH; ^e^ DPPH radical scavenging capacity of PCM; ^f^ DPPH radical scavenging capacity of PCH; ^g^ Lipid peroxidation inhibitory activity of PCM; ^h^ Lipid peroxidation inhibitory activity of PCH; ^i^ Nitric oxide radical scavenging capacity of PCM; ^j^ Nitric oxide radical scavenging capacity of PCH; ^k^ Ferric reducing antioxidant power of PCM; ^l^ Ferric reducing antioxidant power of PCH

**Supplementary Table 4**

*Biological activities of active principles present in phytococktail extracts.*

| **Phyto-chemotypes** | **Biological activity** |
| --- | --- |
| 2-Methoxynaphthalene | Used as an intermediate for the synthesis of nonsteroidal anti-inflammatory drugs (NSAIDs) such as nabumetone [4-(6-methoxy-2-naphthalenyl)-2-butanone] and naproxen, also used for soap perfumes |
| 2-Furancarboxaldehyde, 5-(hydroxymethyl)- | Used in synthesis of dialdehydes, glycols, ethers, amino alcohols and acetals, as an aqueous acid it catalyzes ring opening, could be utilized to produce a wide range of products such as polymers, surfactants, solvents, pharmaceuticals, biofuel, and plant protection agents, inhibits the formation of sickled cells in the blood |
| Eugenol | [Antiestrogenic](http://www.ars-grin.gov/cgi-bin/duke/chemical_activity.pl?Antiestrogenic), [antifeedant](http://www.ars-grin.gov/cgi-bin/duke/chemical_activity.pl?Antifeedant), [antigenotoxic](http://www.ars-grin.gov/cgi-bin/duke/chemical_activity.pl?Antigenotoxic), [antiherpetic](http://www.ars-grin.gov/cgi-bin/duke/chemical_activity.pl?Antiherpetic), [antiinflammatory](http://www.ars-grin.gov/cgi-bin/duke/chemical_activity.pl?Antiinflammatory), [antikeratotic](http://www.ars-grin.gov/cgi-bin/duke/chemical_activity.pl?Antikeratotic), [antimitotic](http://www.ars-grin.gov/cgi-bin/duke/chemical_activity.pl?Antimitotic), [antimutagenic](http://www.ars-grin.gov/cgi-bin/duke/chemical_activity.pl?Antimutagenic),  [antinitrosating](http://www.ars-grin.gov/cgi-bin/duke/chemical_activity.pl?Antinitrosating),  antioxidant, [antioxidant](http://www.ars-grin.gov/cgi-bin/duke/chemical_activity.pl?Antioxidant), [antiprostaglandin](http://www.ars-grin.gov/cgi-bin/duke/chemical_activity.pl?Antiprostaglandin), [antiprostaglandin](http://www.ars-grin.gov/cgi-bin/duke/chemical_activity.pl?Antiprostaglandin), [antipyretic](http://www.ars-grin.gov/cgi-bin/duke/chemical_activity.pl?Antipyretic), [antiradicular](http://www.ars-grin.gov/cgi-bin/duke/chemical_activity.pl?Antiradicular), [insectifuge](http://www.ars-grin.gov/cgi-bin/duke/chemical_activity.pl?Insectifuge), [juvabional](http://www.ars-grin.gov/cgi-bin/duke/chemical_activity.pl?Juvabional),  [larvicide](http://www.ars-grin.gov/cgi-bin/duke/chemical_activity.pl?Larvicide),  [motor-depressant](http://www.ars-grin.gov/cgi-bin/duke/chemical_activity.pl?Motor-Depressant), [nematicide](http://www.ars-grin.gov/cgi-bin/duke/chemical_activity.pl?Nematicide), [neurotoxic](http://www.ars-grin.gov/cgi-bin/duke/chemical_activity.pl?Neurotoxic),  [perfumery](http://www.ars-grin.gov/cgi-bin/duke/chemical_activity.pl?Perfumery),  [pesticide](http://www.ars-grin.gov/cgi-bin/duke/chemical_activity.pl?Pesticide), [prostaglandin-synthesis-inhibitor](http://www.ars-grin.gov/cgi-bin/duke/chemical_activity.pl?Prostaglandin-Synthesis-Inhibitor), [prostaglandin-synthesis-inhibitor](http://www.ars-grin.gov/cgi-bin/duke/chemical_activity.pl?Prostaglandin-Synthesis-Inhibitor), [antisalmonella](http://www.ars-grin.gov/cgi-bin/duke/chemical_activity.pl?Antisalmonella), antiseptic, [antistaphylococcic](http://www.ars-grin.gov/cgi-bin/duke/chemical_activity.pl?Antistaphylococcic),  [antithromboxane](http://www.ars-grin.gov/cgi-bin/duke/chemical_activity.pl?Antithromboxane),  antitumor,  antiulcer,  [antiviral](http://www.ars-grin.gov/cgi-bin/duke/chemical_activity.pl?Antiviral), [apifuge](http://www.ars-grin.gov/cgi-bin/duke/chemical_activity.pl?Apifuge),  [CNS-depressant](http://www.ars-grin.gov/cgi-bin/duke/chemical_activity.pl?CNS-Depressant),  [COX-1-inhibitor](http://www.ars-grin.gov/cgi-bin/duke/chemical_activity.pl?COX-1-Inhibitor), [COX-2-inhibitor](http://www.ars-grin.gov/cgi-bin/duke/chemical_activity.pl?COX-2-Inhibitor), [calcium-antagonist](http://www.ars-grin.gov/cgi-bin/duke/chemical_activity.pl?Calcium-Antagonist), [cancer-preventive](http://www.ars-grin.gov/cgi-bin/duke/chemical_activity.pl?Cancer-Preventive),  [candidicide](http://www.ars-grin.gov/cgi-bin/duke/chemical_activity.pl?Candidicide),  [carminative](http://www.ars-grin.gov/cgi-bin/duke/chemical_activity.pl?Carminative),  [choleretic](http://www.ars-grin.gov/cgi-bin/duke/chemical_activity.pl?Choleretic),  cytochrome-p450-inhibitor,  [cytotoxic](http://www.ars-grin.gov/cgi-bin/duke/chemical_activity.pl?Cytotoxic), [dermatitigenic](http://www.ars-grin.gov/cgi-bin/duke/chemical_activity.pl?Dermatitigenic),  [enterorelaxant](http://www.ars-grin.gov/cgi-bin/duke/chemical_activity.pl?Enterorelaxant),  [flavor](http://www.ars-grin.gov/cgi-bin/duke/chemical_activity.pl?FLavor), [fungicide](http://www.ars-grin.gov/cgi-bin/duke/chemical_activity.pl?Fungicide),  [hepatoprotective](http://www.ars-grin.gov/cgi-bin/duke/chemical_activity.pl?Hepatoprotective), [herbicide](http://www.ars-grin.gov/cgi-bin/duke/chemical_activity.pl?Herbicide),  [insecticide](http://www.ars-grin.gov/cgi-bin/duke/chemical_activity.pl?Insecticide),  [Acaricide](http://www.ars-grin.gov/cgi-bin/duke/chemical_activity.pl?Acaricide), [allergenic](http://www.ars-grin.gov/cgi-bin/duke/chemical_activity.pl?Allergenic),  [analgesic](http://www.ars-grin.gov/cgi-bin/duke/chemical_activity.pl?Analgesic), [anesthetic](http://www.ars-grin.gov/cgi-bin/duke/chemical_activity.pl?Anesthetic), [antiTNF](http://www.ars-grin.gov/cgi-bin/duke/chemical_activity.pl?AntiTNF), [antiaggregant](http://www.ars-grin.gov/cgi-bin/duke/chemical_activity.pl?Antiaggregant), [antiarachidonate](http://www.ars-grin.gov/cgi-bin/duke/chemical_activity.pl?Antiarachidonate),  [antibacterial](http://www.ars-grin.gov/cgi-bin/duke/chemical_activity.pl?Antibacterial), [antibacterial](http://www.ars-grin.gov/cgi-bin/duke/chemical_activity.pl?Antibacterial), [anticonvulsant](http://www.ars-grin.gov/cgi-bin/duke/chemical_activity.pl?Anticonvulsant),  [antiedemic](http://www.ars-grin.gov/cgi-bin/duke/chemical_activity.pl?Antiedemic), [sedative](http://www.ars-grin.gov/cgi-bin/duke/chemical_activity.pl?Sedative),  [termiticide](http://www.ars-grin.gov/cgi-bin/duke/chemical_activity.pl?Termiticide), [trichomonicide](http://www.ars-grin.gov/cgi-bin/duke/chemical_activity.pl?Trichomonicide), [trichomonistat](http://www.ars-grin.gov/cgi-bin/duke/chemical_activity.pl?Trichomonistat), [trypsin-enhancer](http://www.ars-grin.gov/cgi-bin/duke/chemical_activity.pl?Trypsin-Enhancer),  [ulcerogenic](http://www.ars-grin.gov/cgi-bin/duke/chemical_activity.pl?Ulcerogenic), [varroacide](http://www.ars-grin.gov/cgi-bin/duke/chemical_activity.pl?Varroacide),  [vasodilator](http://www.ars-grin.gov/cgi-bin/duke/chemical_activity.pl?Vasodilator),  [vermifuge](http://www.ars-grin.gov/cgi-bin/duke/chemical_activity.pl?Vermifuge), [antispasmodic](http://www.ars-grin.gov/cgi-bin/duke/chemical_activity.pl?Antispasmodic) |
| Methyl-4-hydroxybenzoate | Antioxidant, anti-fungal agent, used in a variety of cosmetics and personal-care products, also used as a food preservative, has a[estrogenic](http://en.wikipedia.org/wiki/Estrogen) effect |
| Aceteugenol | Contains analgesic and antiseptic properties, used in biocides, insect attractants, perfumes, essential oils, and medicines |
| α-D-Glucopyranoside, O-alpha-D-glucopyranosyl-(1.fwdarw.3)-β-D-fructofuranosyl | Preservative |
| Methyl palmitate | Anti-inflammatory, Anti-fibrotic effect, prevents CCl4-induced liver fibrosis linked to reduced TGF-β |
| Palmitoleic acid | Influence fatty liver deposition/production, insulin action, palmitate, and fatty acid synthase by its "[lipokine](http://en.wikipedia.org/wiki/Lipokine)" or hormone-like effects, attenuate hyperglycemia and hypertriglyceridemia by increasing insulin sensitivity, could have a role as a signaling molecule affecting body weight by affecting fat oxidation, 5-alpha-reductase-inhibitor, soap |
| Palmitic acid | Antioxidant, hypocholesterolemic, nematicide, pesticide, lubricant, antiandrogenic, flavor, hemolytic 5-alpha reductase inhibitor, [antialopecic](http://www.ars-grin.gov/cgi-bin/duke/chemical_activity.pl?Antialopecic),   [antifibrinolytic](http://www.ars-grin.gov/cgi-bin/duke/chemical_activity.pl?Antifibrinolytic),  [antioxidant](http://www.ars-grin.gov/cgi-bin/duke/chemical_activity.pl?Antioxidant), [flavor](http://www.ars-grin.gov/cgi-bin/duke/chemical_activity.pl?FLavor), [hemolytic](http://www.ars-grin.gov/cgi-bin/duke/chemical_activity.pl?Hemolytic),  [hypercholesterolemic](http://www.ars-grin.gov/cgi-bin/duke/chemical_activity.pl?Hypercholesterolemic),  [lubricant](http://www.ars-grin.gov/cgi-bin/duke/chemical_activity.pl?Lubricant),  [nematicide](http://www.ars-grin.gov/cgi-bin/duke/chemical_activity.pl?Nematicide),  [pesticide](http://www.ars-grin.gov/cgi-bin/duke/chemical_activity.pl?Pesticide), [propecic](http://www.ars-grin.gov/cgi-bin/duke/chemical_activity.pl?Propecic),  [soap](http://www.ars-grin.gov/cgi-bin/duke/chemical_activity.pl?Soap), used to produce detergents and cosmetics, used in the treatment of schizophrenia |
| Methyl oleate | Used as raw material of emulsifiers or oiling agents for foods, spin finishes and textiles, lubricants for plastics,  paint and ink additives, surfactants and base materials for perfumery, used as solvents or cosolvents, oil carrier in agricultural industry, used as a substitute for methylbenzene, dimethylbenzene, solvent oil, as environmentally-friendly pesticide solvent, used as a synergist of herbicide, as medical intermediate, used to produce various antibiotics, used as a non-fluorescing slurry lubricant, improve adsorbability and wettability of mud cakes and drill metal surface to reduce adhesion coefficient and achieve desirable thermal stability, compatibility and uniform dispersion, used to make cosmetics, detergents, textile assistant |
| Oleic acid | 5-Alpha-reductase-inhibitor, allergenic, anemiagenic, [antialopecic](http://www.ars-grin.gov/cgi-bin/duke/chemical_activity.pl?Antialopecic), [antiandrogenic](http://www.ars-grin.gov/cgi-bin/duke/chemical_activity.pl?Antiandrogenic), [antiinflammatory](http://www.ars-grin.gov/cgi-bin/duke/chemical_activity.pl?Antiinflammatory), antileukotriene-D4; cancer-preventive, choleretic, [dermatitigenic](http://www.ars-grin.gov/cgi-bin/duke/chemical_activity.pl?Dermatitigenic), flavor, hypocholesterolemic, insectifuge, irritant, percutaneostimulant, perfumery, propecic, Activates protein kinase C in hepatocytes, uncouples oxidative phosphorylation, inhibits 2,4-dinitrophenol-stimulated ATPase |
| Piperine | AHH-inhibitor, anticlastogen, anticonvulsant, antiedemic, antifertility, antiglucuronidase, antiimplantation,  antiinflammatory, antileishmanic,  antimutagenic, antinarcotic, antinociceptive,  antioxidant,  antiplasmodial,  antipyretic, antiseptic,  antispasmodic, aryl-hydrocarbon-hydroxylase-inhibitor, diaphoretic,  epinephrininergic,  flavor, [hepatoprotective](http://www.ars-grin.gov/cgi-bin/duke/chemical_activity.pl?Hepatoprotective), hepatoregenerative, hypertensive, [hypotensive](http://www.ars-grin.gov/cgi-bin/duke/chemical_activity.pl?Hypotensive), insecticide,  [secretogogue](http://www.ars-grin.gov/cgi-bin/duke/chemical_activity.pl?Secretogogue),  [serotoninergic](http://www.ars-grin.gov/cgi-bin/duke/chemical_activity.pl?Serotoninergic),  spermigenic, stimulant,  [thermogenic](http://www.ars-grin.gov/cgi-bin/duke/chemical_activity.pl?Thermogenic), [thyrostimulant](http://www.ars-grin.gov/cgi-bin/duke/chemical_activity.pl?Thyrostimulant), cancer preventive, [endorphinogenic](http://www.ars-grin.gov/cgi-bin/duke/chemical_activity.pl?Endorphinogenic), peristaltic, pesticide, analgesic, anesthetic, [antisubstance-P](http://www.ars-grin.gov/cgi-bin/duke/chemical_activity.pl?AntiSubstance-P), [parasiticide](http://www.ars-grin.gov/cgi-bin/duke/chemical_activity.pl?Parasiticide), CNS-stimulant,  cardiotonic, carminative, catecholaminogenic, abortifacient, adrenergic,  analeptic,  antiaflatoxin,  antibacterial, ATPase stimulant, inhibit human CYP3A4 and P-glycoprotein,  increase the bioavailability of various compounds like [curcumin](http://en.wikipedia.org/wiki/Curcumin), have chemopreventive efficacy, stimulate pigmentation in the skin, have anti-depression like activity, and cognitive enhancing effects, anti-arthritic |
| α-Tocopherol | Antiinflammatory, antiischemic, antileukemic, antileukotriene, [antilithic](http://www.ars-grin.gov/cgi-bin/duke/chemical_activity.pl?Antilithic), [antilupus](http://www.ars-grin.gov/cgi-bin/duke/chemical_activity.pl?Antilupus), [antimaculitic](http://www.ars-grin.gov/cgi-bin/duke/chemical_activity.pl?Antimaculitic),  [antimastalgic](http://www.ars-grin.gov/cgi-bin/duke/chemical_activity.pl?Antimastalgic),  [antimelanomic](http://www.ars-grin.gov/cgi-bin/duke/chemical_activity.pl?Antimelanomic), antimyoclonic,  [antirheumatic](http://www.ars-grin.gov/cgi-bin/duke/chemical_activity.pl?Antirheumatic), [antisenility](http://www.ars-grin.gov/cgi-bin/duke/chemical_activity.pl?Antisenility), [antisickling](http://www.ars-grin.gov/cgi-bin/duke/chemical_activity.pl?Antisickling), antispasmodic, antisterility,  antistroke,  antisunburn,  [antisyndrome-X](http://www.ars-grin.gov/cgi-bin/duke/chemical_activity.pl?Antisyndrome-X), antithalassemic, antithrombotic, [antithromboxane-B2](http://www.ars-grin.gov/cgi-bin/duke/chemical_activity.pl?Antithromboxane-B2), [antitoxemic](http://www.ars-grin.gov/cgi-bin/duke/chemical_activity.pl?Antitoxemic), [antitumor](http://www.ars-grin.gov/cgi-bin/duke/chemical_activity.pl?Antitumor); [antitumor (breast)](http://www.ars-grin.gov/cgi-bin/duke/chemical_activity.pl?Antitumor (Breast)), [antitumor (colorectal)](http://www.ars-grin.gov/cgi-bin/duke/chemical_activity.pl?Antitumor (Colorectal)), [calcium-antagonist](http://www.ars-grin.gov/cgi-bin/duke/chemical_activity.pl?Calcium-Antagonist), [cancer-preventive](http://www.ars-grin.gov/cgi-bin/duke/chemical_activity.pl?Cancer-Preventive),  [cardioprotective](http://www.ars-grin.gov/cgi-bin/duke/chemical_activity.pl?Cardioprotective),  [cerebroprotective](http://www.ars-grin.gov/cgi-bin/duke/chemical_activity.pl?Cerebroprotective), [circulatory-stimulant](http://www.ars-grin.gov/cgi-bin/duke/chemical_activity.pl?Circulatory-Stimulant),  [circulotonic](http://www.ars-grin.gov/cgi-bin/duke/chemical_activity.pl?Circulotonic), [hepatoprotective](http://www.ars-grin.gov/cgi-bin/duke/chemical_activity.pl?Hepatoprotective), 5-HETE-inhibitor, allergenic, analgesic, antiMD, antiMS, antiPMS, antiaggregant,  antiaging, antialzheimeran, antianginal, antiarteriosclerotic, antiarthritic, antiatherosclerotic, antibronchitic, anticancer (breast), anticariogenic,  [anticataract](http://www.ars-grin.gov/cgi-bin/duke/chemical_activity.pl?Anticataract), [antichorea](http://www.ars-grin.gov/cgi-bin/duke/chemical_activity.pl?Antichorea), [antichoreic](http://www.ars-grin.gov/cgi-bin/duke/chemical_activity.pl?Antichoreic), [antidysmenorrheic](http://www.ars-grin.gov/cgi-bin/duke/chemical_activity.pl?Antidysmenorrheic),  antiepitheleomic,  [antifibrositic](http://www.ars-grin.gov/cgi-bin/duke/chemical_activity.pl?Antifibrositic),  [antiglycosation](http://www.ars-grin.gov/cgi-bin/duke/chemical_activity.pl?Antiglycosation),  [antiherpetic](http://www.ars-grin.gov/cgi-bin/duke/chemical_activity.pl?Antiherpetic),  [antiinfertility](http://www.ars-grin.gov/cgi-bin/duke/chemical_activity.pl?Antiinfertility),  [hypocholesterolemic](http://www.ars-grin.gov/cgi-bin/duke/chemical_activity.pl?Hypocholesterolemic), [hypoglycemic](http://www.ars-grin.gov/cgi-bin/duke/chemical_activity.pl?Hypoglycemic), [immunomodulator](http://www.ars-grin.gov/cgi-bin/duke/chemical_activity.pl?Immunomodulator), [immunostimulant](http://www.ars-grin.gov/cgi-bin/duke/chemical_activity.pl?Immunostimulant), [insulin-sparing](http://www.ars-grin.gov/cgi-bin/duke/chemical_activity.pl?Insulin-Sparing), [lipoxygenase-inhibitor](http://www.ars-grin.gov/cgi-bin/duke/chemical_activity.pl?Lipoxygenase-Inhibitor), [NO-inhibitor](http://www.ars-grin.gov/cgi-bin/duke/chemical_activity.pl?NO-Inhibitor), [ornithine-decarboxylase-inhibitor](http://www.ars-grin.gov/cgi-bin/duke/chemical_activity.pl?Ornithine-Decarboxylase-Inhibitor), [P21-inducer](http://www.ars-grin.gov/cgi-bin/duke/chemical_activity.pl?P21-Inducer), antidecubitic,  antidementia,  [antidermatitic](http://www.ars-grin.gov/cgi-bin/duke/chemical_activity.pl?Antidermatitic), [antidiabetic](http://www.ars-grin.gov/cgi-bin/duke/chemical_activity.pl?Antidiabetic), [phospholipase-A2-inhibitor](http://www.ars-grin.gov/cgi-bin/duke/chemical_activity.pl?Phospholipase-A2-Inhibitor), [protein-kinase-C-inhibitor](http://www.ars-grin.gov/cgi-bin/duke/chemical_activity.pl?Protein-Kinase-C-Inhibitor), [vasodilator](http://www.ars-grin.gov/cgi-bin/duke/chemical_activity.pl?Vasodilator), anticonvulsant, anticoronary, antitumor (prostate), [antineuritic](http://www.ars-grin.gov/cgi-bin/duke/chemical_activity.pl?Antineuritic), [antineuropathic](http://www.ars-grin.gov/cgi-bin/duke/chemical_activity.pl?Antineuropathic),  [antinitrosaminic](http://www.ars-grin.gov/cgi-bin/duke/chemical_activity.pl?Antinitrosaminic),  [antiophthalmic](http://www.ars-grin.gov/cgi-bin/duke/chemical_activity.pl?Antiophthalmic), [antiosteoarthritic](http://www.ars-grin.gov/cgi-bin/duke/chemical_activity.pl?Antiosteoarthritic), [antioxidant](http://www.ars-grin.gov/cgi-bin/duke/chemical_activity.pl?Antioxidant), [antiparkinsonian](http://www.ars-grin.gov/cgi-bin/duke/chemical_activity.pl?Antiparkinsonian), [antiproliferant](http://www.ars-grin.gov/cgi-bin/duke/chemical_activity.pl?Antiproliferant), [antiradicular](http://www.ars-grin.gov/cgi-bin/duke/chemical_activity.pl?Antiradicular), [antiretinopathic](http://www.ars-grin.gov/cgi-bin/duke/chemical_activity.pl?Antiretinopathic?), [antitumor (stomach)](http://www.ars-grin.gov/cgi-bin/duke/chemical_activity.pl?Antitumor (Stomach)), [antiulcerogenic](http://www.ars-grin.gov/cgi-bin/duke/chemical_activity.pl?Antiulcerogenic), [apoptotic](http://www.ars-grin.gov/cgi-bin/duke/chemical_activity.pl?Apoptotic) |
| τ-Sitosterol | Febrifuge, [gonadotrophic](http://www.ars-grin.gov/cgi-bin/duke/chemical_activity.pl?Gonadotrophic), [hepatoprotective](http://www.ars-grin.gov/cgi-bin/duke/chemical_activity.pl?Hepatoprotective), [hypocholesterolemic](http://www.ars-grin.gov/cgi-bin/duke/chemical_activity.pl?Hypocholesterolemic), [antiedemic](http://www.ars-grin.gov/cgi-bin/duke/chemical_activity.pl?Antiedemic), [antiestrogenic](http://www.ars-grin.gov/cgi-bin/duke/chemical_activity.pl?Antiestrogenic), [antifeedant](http://www.ars-grin.gov/cgi-bin/duke/chemical_activity.pl?Antifeedant),  [hypocholesterolemic](http://www.ars-grin.gov/cgi-bin/duke/chemical_activity.pl?Hypocholesterolemic), hypoglycemic, [hypolipidemic](http://www.ars-grin.gov/cgi-bin/duke/chemical_activity.pl?Hypolipidemic), [antiinflammatory](http://www.ars-grin.gov/cgi-bin/duke/chemical_activity.pl?Antiinflammatory),  [antileukemic](http://www.ars-grin.gov/cgi-bin/duke/chemical_activity.pl?Antileukemic), [antilymphomic](http://www.ars-grin.gov/cgi-bin/duke/chemical_activity.pl?Antilymphomic),  antiviral, apoptotic, [artemicide](http://www.ars-grin.gov/cgi-bin/duke/chemical_activity.pl?Artemicide), caspase-8-inducer, estrogenic, androgenic, angiogenic,  anorexic,  antiadenomic,  antiandrogenic, antibacterial, anticancer (breast), anticancer (cervix), anticancer (lung),  antifertility,  antigonadotrophic, pesticideantihyperlipoproteinaemic, cancer-preventive, candidicide |
| Cuminic aldehyde | Daily Flavor, food Flavor, inhibition of pieris rapae phenoloxidase, inhibitory activity was evaluated against the oxidation of L-3,4-dihydroxyphenylalanine (L-DOPA) catalyzed by mushroom tyrosinase, inhibition of human FAAH (fatty-acid amide hydrolase 1) |
| Trans-Caryophyllene | Anti-tumor, analgesic**,** antibacterial, antiinflammatory, sedative, fungicide |
| α-Humulene | anti-inflammatory properties, decrease the edema formation by acting as dexamethazone, inhibitory effects on tumor necrosis factor-α (TNFα) and interleukin-1 β (IL1B) generation |
| ar-Curcumene | [Antirhinoviral](http://www.ars-grin.gov/cgi-bin/duke/chemical_activity.pl?Antirhinoviral), antiulcer, antiviral |
| Caryophyllene oxide | Antiedemic, antifeedant, antiinflammatory, antitumor, calcium-antagonist, fungicide, insecticide |
