# Supplementary figures and images for: Chemical composition and antioxidant capacities of phytococktail extracts from trans-Himalayan cold desert
Source: BMC Complement Altern Med. 2013 Oct 7;13:259. doi: 10.1186/1472-6882-13-259 (PMC3854071; doi:10.1186/1472-6882-13-259)

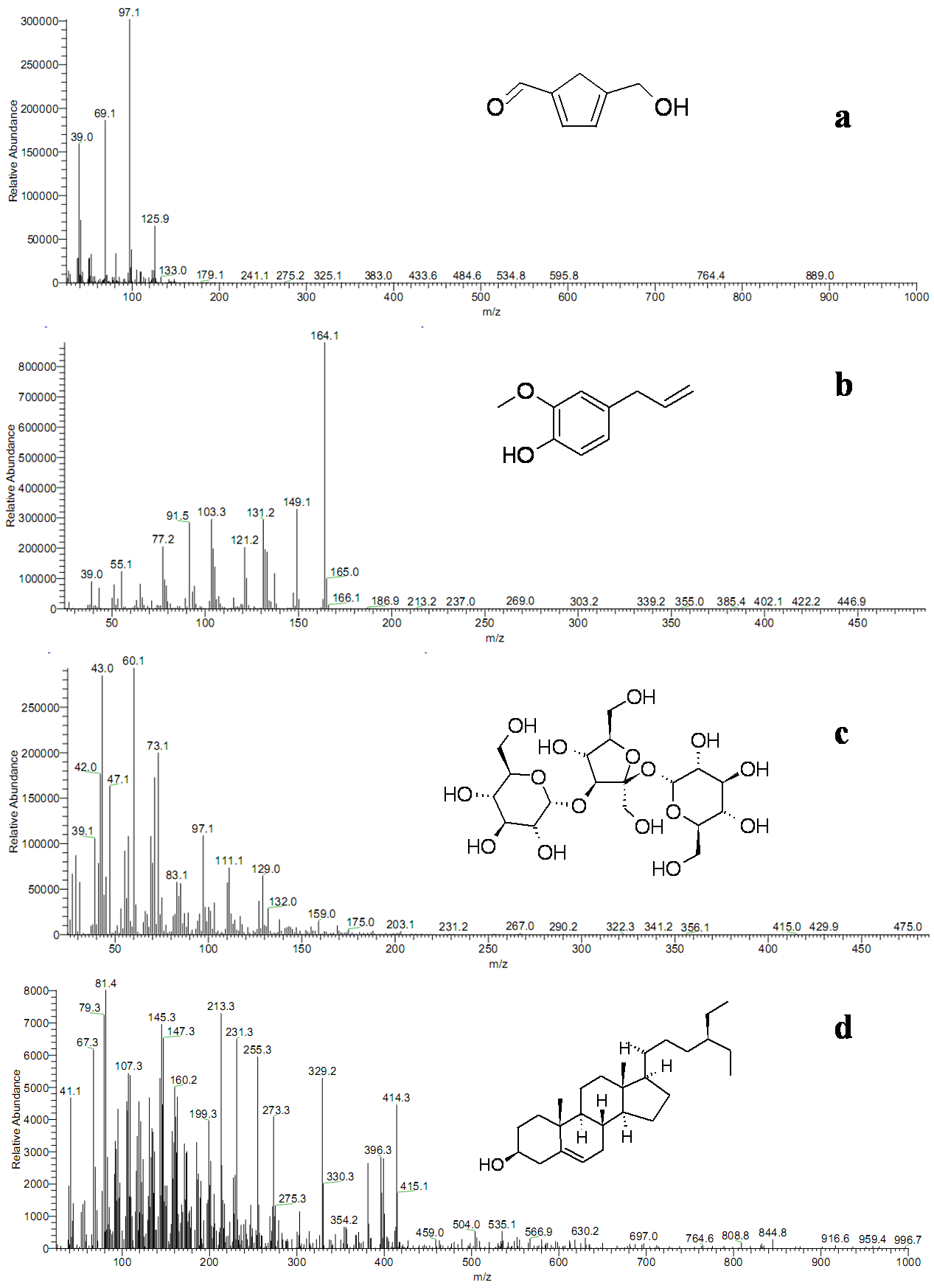

Supplement: Additional file 2: Figure S1 — Relative abundance of major compounds in methanol extract. a. 2-Furancarboxaldehyde, 5-(hydroxymethyl)- , b. Eugenol, c. α-D-glucopyranoside, O-α-D-glucopyranosyl-(1.fwdarw.3)-β-D-fructofuranosyl, d. τ-Sitosterol. [file 1472-6882-13-259-S2.tiff]

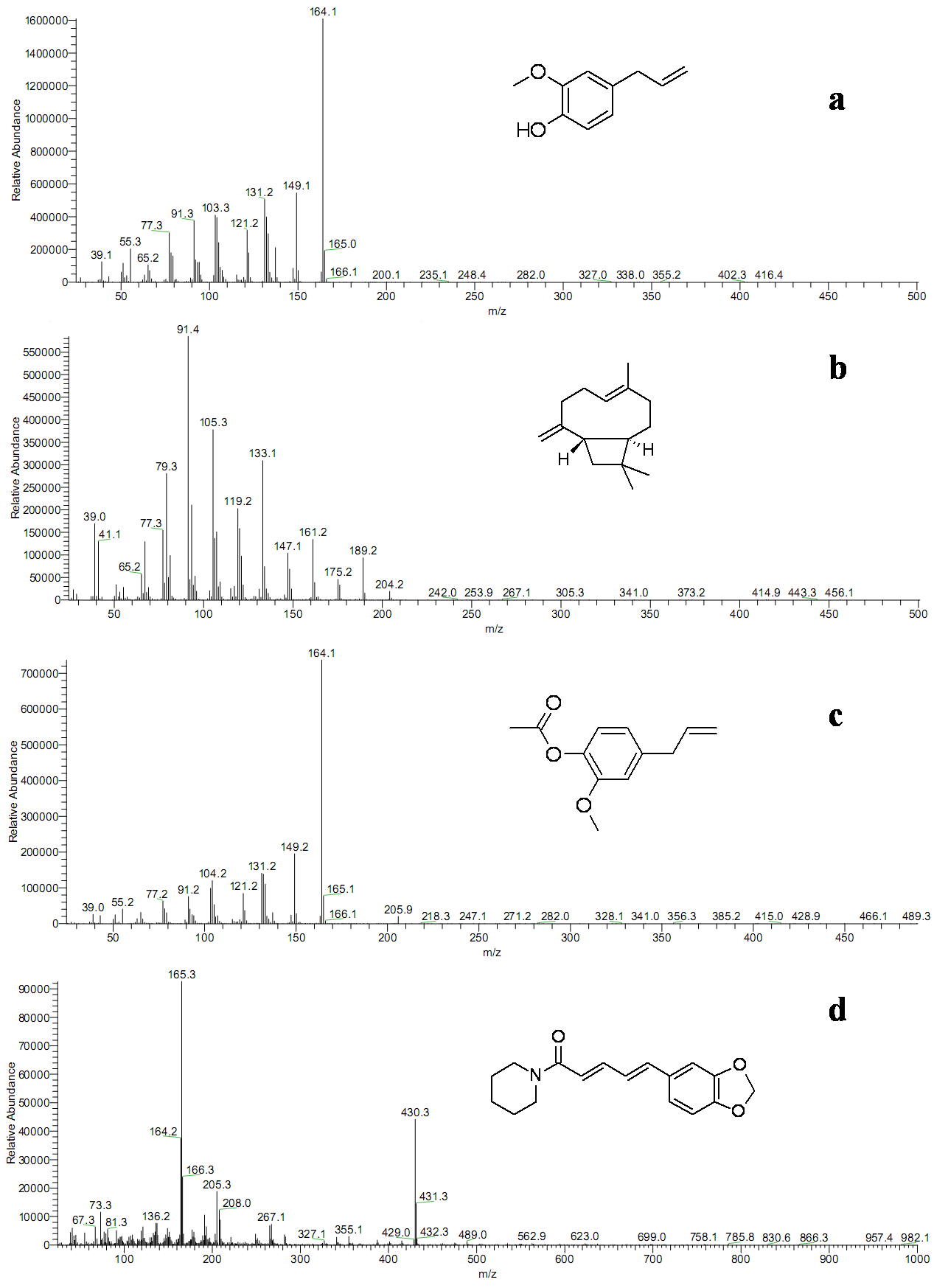

Supplement: Additional file 3: Figure S2 — Relative abundance of major compounds in n-hexane extract. a. Eugenol, b. trans-Caryophyllene, c. Aceteugenol, d. Piperine. [file 1472-6882-13-259-S3.tiff]
